# A framework for performance enhancement of classifiers in detection of prostate cancer from microarray gene

**DOI:** 10.1016/j.heliyon.2024.e29630

**Published:** 2024-04-25

**Authors:** Kalaiyarasi Mani, Harikumar Rajaguru

**Affiliations:** Bannari Amman Institute of Technology, India

**Keywords:** Prostate cancer (PCa), Microarray gene data, Adaboost, XGboost, Elephant Herding Optimization, Harmonic search, Quadrature discriminant analysis

## Abstract

Prostate cancer is a major world health problem for men. This shows how important early detection and accurate diagnosis are for better treatment and patient outcomes. This study compares different ways to find Prostate Cancer (PCa) and label tumors as normal or abnormal, with the goal of speeding up current work in microarray gene data analysis. The study looks at how well several feature extraction methods work with three feature selection strategies: Harmonic Search (HS), Firefly Algorithm (FA), and Elephant Herding Optimization (EHO). The techniques tested are Expectation Maximization (EM), Nonlinear Regression (NLR), K-means, Principal Component Analysis (PCA), and Discrete Cosine Transform (DCT). Eight classifiers are used for the task of classification. These are Random Forest, Decision Tree, Adaboost, XGBoost, and Support Vector Machine (SVM) with linear, polynomial, and radial basis function kernels. This study looks at how well these classifiers work with and without feature selection methods. It finds that the SVM with radial basis function kernel, using DCT for feature extraction and EHO for feature selection, does the best of all of them, with an accuracy of 94.8 % and an error rate of 5.15 %.

## 1 Introduction

Cancer poses a significant and unappreciated risk to public health. There is no expedient solution for cancer. Timely identification of cancer results in an improved forecast and an extended lifespan. Prostate cancer (PCa) poses a significant challenge due to its varied progression rates and the difficulty in distinguishing aggressive from non-aggressive forms. Traditional diagnostic methods, such as the Gleason method, suffer from subjectivity and low reproducibility among observers. This leads to challenges in accurately predicting disease progression and determining suitable treatment strategies. The occurrence and death rates of Prostate Cancer (PCa) are directly related to advancing age [1]. Prostate cancer (PCa) is the second most common malignancy in males around the world. A precise diagnosis of PCa is difficult to achieve for the simple reason that it is critical to determine which cases of PCa are destined to advance and which cases could benefit from early, drastic treatment [2]. During the preceding year 2022, the estimated number of new cases was 268,490, and the rate of mortality was 34,500. According to the latest global cancer statistics, the most frequently diagnosed cancer in males is lung cancer, accounting for 14.5 % of cases, followed by Prostate Cancer (PCa) at 13.5 %. Prostate Cancer (PCa) has the highest incidence rate among men in over 100 countries [3,4].

To our good fortune, the majority of prostate tumors tend to develop slowly and are of the low-grade variety, which has a relatively modest risk and exhibits a limited degree of severity. In the majority of instances, there are no early or initial symptoms [5]. However, late symptoms may include exhaustion caused by anemia, pain in the bones, paralysis caused by spinal metastases, and renal failure caused by bilateral ureteral obstruction. Pathologists can use the Gleason method to score prostate cancer (PCa) tissue microarrays under numerous tissue microarray images; however, this is a time-consuming process that is prone to fluctuation among various observers and has low reproducibility [6].

Cancer, a widespread and frequently underestimated danger to the well-being of the population, can be thoroughly examined via genetic testing, specifically targeting Prostate Cancer (PCa). The advent of DNA microarray technology offers a promising approach to explore alterations in the genomic DNA sequence within cancer cells. This novel methodology allows for the concurrent analysis of numerous genes, providing a full comprehension of gene expression and possible alterations linked to PCa [5].

Microarray technology is employed in functional genomics to analyze data from many research endeavors, enabling the examination of gene expression alterations in sick tissues and yielding valuable insights. Hybridization techniques enable the simultaneous monitoring of numerous genes, rendering microarrays highly useful in cancer research, medication development, disease detection, and environmental engineering [6]. Gene expression has a significant role in determining the protein composition of a cell, which in turn affects the cell's responsiveness and ability to operate. Genes serve as the definitive determinants of an organism's traits [7]. Scientists utilize microarrays to monitor the quantities of DNA or RNA over a period of time, assisting in the detection of underlying factors responsible for consistent variations observed in cancer experiments. The process of microarray technology entails the application of a fluorescent dot onto an array blade for each individual gene, followed by the subsequent creation, preparation, and analysis of the microarrays. In a two-color microarray experiment, the fluorescent patch of each gene is compared to a reference image, and the intensity of fluorescent light is evaluated to estimate the levels of gene expression. The process of extracting point intensity features and normalizing data is crucial in defining microarray spots. This allows for a comprehensive understanding of gene expression at various time points or in diverse experimental conditions [8,9].

Microarrays, due to their capacity to evaluate alterations in gene expression and identify possible biomarkers, provide a more advanced approach for detecting and classifying Prostate Cancer. The combination of this technology with advancements in data processing and normalization shows great potential in enhancing our comprehension of cancer biology and optimizing diagnostic and therapeutic approaches for the betterment of public health [[5], [6], [7], [8], [9]].

This research is necessary to improve the accuracy and reliability of prostate cancer detection using microarray gene expression data. The proposed approach combines multiple classifiers and ensemble learning techniques to enhance performance. By addressing the challenges of high dimensionality and biological variability, this research aims to provide more accurate and timely diagnoses, ultimately improving patient outcomes. The findings may have broader implications for cancer detection and classification in other types of cancer as well.

The aim of this research is to develop a system that accurately categorizes Prostate Cancer (PCa) and organizes cancer data in a way that facilitates treatment for patients and reduces risks. However, inaccurate data caused by irrelevant or noisy genes can reduce the accuracy of the classifier. Therefore, efficient gene selection and feature extraction methods are necessary to choose informative features. Multiple procedures are also needed to achieve a perfect classification of cancer data and improve classification accuracy.

Data analysis techniques, such as feature extraction, feature selection, and classification algorithms, play a vital role in making sense of complex microarray data. These techniques help in identifying informative genes, reducing noise, and improving the accuracy of cancer classification models. By integrating sophisticated data analysis techniques with genetic testing, researchers can enhance the accuracy and reliability of prostate cancer diagnosis, ultimately leading to improved patient outcomes. The proposed research aims to address current challenges in prostate cancer management by leveraging advanced data analysis techniques and genetic testing. The potential impact includes more accurate and timely diagnoses, personalized treatment strategies, and advancements in cancer research. By improving the accuracy and reliability of prostate cancer diagnosis, the research contributes to better patient outcomes and reduced risks associated with misdiagnosis or delayed diagnosis.

Five feature extraction strategies are used to choose interesting genes and improve microarray high-dimension data classification accuracy. Eight classifiers categorize features as normal or abnormal after three feature selection methods are used. Researchers are creating more accurate decision support models to take advantage of this technology, which has been used to forecast prostate cancer (PCa). This research also underlines the necessity for a computer-aided technique to classify ovarian cancer from microarray gene expression to aid medical and clinical specialists in diagnosis. Classification accuracy is excellent in this work. The methodologies developed in this research have broader implications beyond prostate cancer, with potential applications in other types of cancer diagnosis and classification. The integration of advanced data analysis techniques and machine learning algorithms can lead to advancements in oncology and healthcare delivery on a larger scale. The research contributes to optimizing treatment plans, personalized medicine approaches, and overall advancements in cancer research and healthcare.

## 2 Related works

Prostate Cancer (PCa) is a major health issue for men worldwide. Microarray gene expression data has been widely used for Prostate Cancer (PCa) classification, as it allows for the identification of genetic biomarkers that can distinguish between different stages of the disease. Several studies have been conducted to develop accurate and reliable classification models for Prostate Cancer (PCa) using microarray gene expression data. Here is a brief overview of some of the key findings from these studies.

Zhang et al. (2019) used a support vector machine (SVM) model using microarray gene expression data from Prostate Cancer (PCa) patients. They found that their model achieved an accuracy of 95.4 % in distinguishing between cancerous and non-cancerous samples [10]. Xiao et al. (2019) used a random forest algorithm to classify Prostate Cancer (PCa) samples based on microarray gene expression data. They found that their model achieved an accuracy of 89.2 % in distinguishing between cancerous and non-cancerous samples [11]. However, while these studies showcase promising results, there remains a need for more robust validation techniques to ensure the reliability of these models across diverse patient populations.

Bhagirath et al. (2020) developed a logistic regression model to predict Prostate Cancer (PCa) recurrence based on microarray gene expression data. They found that their model achieved an accuracy of 86.4 % in predicting recurrence [12]. Despite these advancements, limitations such as the lack of consideration for potential confounding factors and the limited exploration of novel biomarkers hinder the broader applicability of these findings. Xu et al. (2020) developed a deep learning model to classify Prostate Cancer (PCa) samples based on microarray gene expression data. They found that their model achieved an accuracy of 92.2 % in distinguishing between cancerous and non-cancerous samples [13] yet further research is needed to address the interpretability and generalizability of deep learning models in clinical settings.

Lee et al. (2019) developed a Bayesian network model to predict Prostate Cancer (PCa) recurrence based on microarray gene expression data. They found that their model achieved an accuracy of 84.6 % in predicting recurrence [14]. Jiang et al. (2015) used a combination of feature selection and SVM to classify Prostate Cancer (PCa) based on microarray gene expression data. The study reported an overall accuracy of 91.1 % in classifying Prostate Cancer (PCa) based on gene expression patterns [15]. However, challenges persist in identifying robust biomarkers and elucidating the underlying biological mechanisms driving PCa progression.

Long et al. (2017) used a combination of feature selection and random forest to classify Prostate Cancer (PCa) based on microarray gene expression data. The study reported an overall accuracy of 92.5 % in classifying Prostate Cancer (PCa) based on gene expression patterns underscoring the potential of feature selection techniques in improving model performance [16]. Wu et al. (2019) used a combination of gene expression data and clinical information to classify Prostate Cancer (PCa) into subtypes. The study used a hierarchical clustering approach to identify four distinct subtypes of Prostate Cancer (PCa) with different clinical outcomes [17]. Despite this progress, the integration of multi-omics data and the development of interpretable models remain areas for future exploration.

Ghosh, S. et al. (2018) used deep learning methods to classify Prostate Cancer (PCa) based on gene expression data. The study reported an overall accuracy of 91.6 % in classifying Prostate Cancer (PCa) based on gene expression patterns [18]. However, challenges such as model interpretability and data heterogeneity require further investigation to enhance the clinical utility of deep learning approaches.

Khavanin Zadeh et al. (2020) compared the performance of various multi-class classification algorithms in classifying Prostate Cancer (PCa) based on gene expression data [19]. The study reported that decision tree algorithms performed the best in classifying Prostate Cancer (PCa) based on gene expression patterns. Nonetheless, the development of robust and interpretable classification models remains an ongoing challenge in the field.

In addition, recent work by Ref. [20] introduced a comprehensive supervised classification approach using Support Vector Machine (SVM) models to investigate differentially expressed Y-chromosome genes in PCa, achieving an average accuracy of 98.3 % across eight SVM models. While this study provides valuable insights into potential biomarkers for PCa, further validation and replication studies are warranted to confirm these findings.

In the realm of prostate cancer (PCa) classification using microarray gene expression data, current studies have shown progress but face limitations. While achieving high accuracy in discerning cancerous from non-cancerous samples, these models lack validation techniques for broader patient populations. There's also a shortfall in exploring new biomarkers and considering potential confounding factors. Challenges lie in the interpretability and generalizability of deep learning models, alongside a need for deeper insights into the biological mechanisms driving PCa progression. Existing studies have insufficiently integrated multi-omics data and explored additional clinical variables, limiting predictive accuracy. The reliance on certain algorithms may restrict model complexity and accuracy. To address these gaps and enhance PCa classification's clinical utility, a new algorithm is needed, emphasizing robust validation, multi-omics data integration, consideration of clinical variables, and improved interpretability.

Overall, these studies demonstrate the potential of microarray gene expression data for Prostate Cancer (PCa) classification and prediction. However, more research is needed to further improve the accuracy and reliability of these models, as well as to identify additional biomarkers that can help improve diagnosis and treatment of the disease.

## 3 Materials and methods

**Fig. 1:**
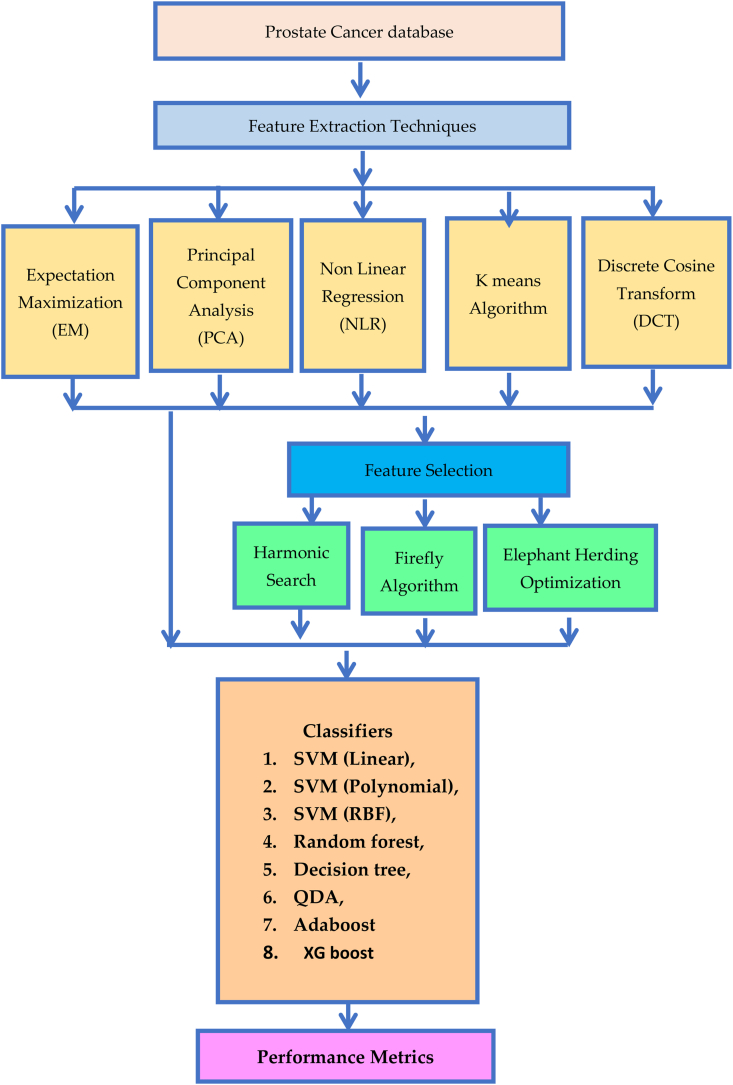
Proposed methodology of Prostate and Normal gene data classification.

The microarray datasets include a wealth of genomic data that, if properly analyzed, might profoundly alter the fields of science and medicine. In order to learn more about the genetic factors that cause cancer and how to treat cancer patients more effectively, microarray tests have been conducted. Developing machine-learning methods for the processing of microarray data has required substantial effort over the past decade. There are a variety of methods for determining whether or not a sample contains malignant cells. Fig. 1 depicts graphically the proposed work.

The 12,600 microarray gene data was dimensionally reduced using extraction techniques such as Expectation Maximization (EM), Principal Component Analysis (PCA), Non Linear Regression (NLR), K means, and Discrete Cosine Transform (DCT) methods. After feature extraction, the features are selected using the Harmonic Search (HS), Firefly Algorithm (FA), and Elephant Herding Optimization (EHO) algorithms. Then the selected features are classified using the eight different classifiers. Based on the classifier results, the performance is analyzed with and without feature selection methods.

Compared to Deep Learning models, the chosen techniques (EM, NLR, K-means, PCA, DCT) are simpler and offer better interpretability. This means researchers can understand what features are most important for classification (e.g., with PCA they can see which principal components contribute most). This is crucial in a medical setting where understanding why a model makes a decision is vital. Microarray gene data analysis often deals with datasets that might be limited in size. expand_more Deep learning models typically require vast amounts of data for optimal performance. expand_more. The chosen techniques are generally less computationally expensive compared to Deep Learning models. This allows for faster training and analysis, which can be beneficial in a clinical setting.

This study aims to compare feature extraction and selection methods, not to achieve the absolute highest accuracy. The chosen techniques provide a good foundation for this comparison while remaining interpretable and efficient. However, careful consideration of interpretability and data size limitations would still be necessary when implementing Deep Learning for prostate cancer classification. Overall, the selection of techniques prioritizes interpretability, efficiency, and facilitating the comparison between feature extraction and selection methods, making them suitable for this specific study.

Our study utilizes microarray gene data in a novel method for diagnosing prostate cancer. Unlike previous research that examined one method at a time, our approach carefully compares and integrates feature extraction, feature selection, and classification methods within a single framework. This thorough analysis enhances the accuracy and efficiency of prostate cancer diagnosis and provides valuable insights for future research. Our work contributes to the field by combining and contrasting various approaches for using microarray gene data in prostate cancer diagnosis. While prior research has primarily focused on individual techniques, our method evaluates and utilizes feature extraction, feature selection, and classification methods together. This approach not only improves diagnostic accuracy and speed but also generates important new ideas for future study in this area.

### 3.1 Microarray gene dataset

Numerous institutions, like the National Center for Biotechnology Information (NCBI), Stanford University, and the European Bioinformatics Institute, offer unrestricted access to genetic databases (EBI). For the purpose of this inquiry, data collection using a Prostate Cancer (PCa) microarray was performed. The microarray Prostate Cancer (PCa) data collection is available to the public [21] and is utilized in this research. This study utilized a total of 136 samples of Prostate Cancer (PCa), consisting of 59 healthy samples and 77 samples that had malignant Prostate Cancer (PCa). Table 1 gives details about the samples used in this research.

**Table 1:** Prostate dataset details.

	Healthy samples	Malignant Prostate Cancer	Total Samples
No. of samples	59	77	136

Microarrays assess thousands of gene expression levels, producing high-dimensional data. Data processing and gene visualization are tough due to the many features. Feature extraction removes features while keeping the dataset's most relevant data. Thus, machine learning algorithms detect data patterns and relationships better. Feature extraction and dimensionality reduction enhance data significance. Feature extraction pulls the most important facts from source data for machine learning algorithms. Dimensionality reduction takes the most significant facts and expresses them in fewer dimensions. Dimensionality reduction reduces features, yet feature extraction adds useful ones.

### 3.2 Feature extraction techniques

Dimensionality reduction is subdivided into feature extraction in pattern recognition and image processing. When an algorithm's input data is too vast to process and is thought to be redundant (lots of data but little information), the input data are transformed to a lower-dimensional format (also known as a features vector). The process of translating input data into a set of features is known as feature extraction. It is anticipated that the required information can be derived from the extracted features if the features are carefully chosen.

A lot of memory and processing power are usually needed for an analysis with a lot of variables. It could also make a classification algorithm fit training samples too well and not fit new samples as well. So, feature extraction helps get the best feature out of those big data sets by picking and combining variables into features. This cuts down on how much data needs to be looked at. These things are not hard to figure out. Less data means less work for the machine to do when building the model. It also speeds up the machine learning steps of learning and generalising. Methods like Expectation Maximization (EM), Non Linear Regression (NLR), K means, Principal Component Analysis (PCA), and Discrete Cosine Transform (DCT) are used to pull out features from prostate gene data.

#### 3.2.1 Expectation Maximization

Expectation Maximization (EM) is an algorithm used in statistical modeling for finding maximum likelihood estimates of parameters in probabilistic models where the data may be incomplete or contain hidden variables. EM can also be used as a feature extraction technique, where the hidden variables can be used as features for subsequent analysis [22]. Convergence in EM is achieved by repeatedly switching between two phases, called the expectation (E) step and the maximization (M) step. Given the data and the current parameter estimations, the method determines the probability distribution of the hidden variables in the E-step. The procedure then proceeds to the M-step, where the parameter estimates are revised in light of the E-calculated step's probability distribution of the hidden variables.

The EM algorithm can be summarized using the following steps:

Initialization: Choose initial values for the parameters of the probabilistic model.

E-step: In the E-step, the posterior probabilities of each data point i belonging to each mixture component j are calculated:

(1) $$w_{ij}=P\left(\left(Z_{j}=1|x_{i},\theta \right)\right)$$

where:

• wij is the posterior probability of data point i belonging to mixture component j
• zj is an indicator variable that takes the value 1 if data point i belongs to mixture component j, and 0 otherwise.
• xi is the feature vector of data point i
• θ is the parameter set, including the mean, covariance, and mixing coefficients of each mixture component

The posterior probability can be calculated using Bayes' theorem:

(2) $$w_{ij}=P\left(\left(x_{i}|z_{j}=1,\theta \right)\right)*P\left(\left(Z_{j}=1|\theta \right)\right)/P\left(\left(x_{i}|\theta \right)\right)$$

where:

• P(xi|zj = 1, θ) is the probability density function of the Gaussian distribution j evaluated at data point i
• P(zj = 1|θ) is the mixing coefficient of the j-th mixture component
• P(xi|θ) is the marginal likelihood of data point i

M-step:

Maximizing the expected log-likelihood of the data with respect to is how the M-step updates the parameter set. This involves updating the mean, covariance, and mixing coefficient of each mixture component.

Update the mixing coefficients.

(3) $$\pi_{j}=\sum_{i}w_{ij}/N$$

where, πj is the mixing coefficient of mixture component j, N is the total number of data points.

Update the mean:

(4) $$\mu_{j}=\sum_{i}w_{\text{ij}}*\;x_{i}/\sum_{i}w_{\text{ij}}$$

where, μj is the mean of mixture component j.

Update the covariance:

(5) $$\sum_{j}=\frac{\sum_{i}{(w_{ij}*\;\left(x_{i}-\mu_{j}\right)\left(x_{i}-\mu_{j}\right)}^{T})}{\sum_{i}w_{ij}}$$

where, Σj is the covariance matrix of mixture component j. These steps are iterated until convergence is reached, i.e., until the log-likelihood of the data no longer increases.

Convergence: Repeat steps until convergence is reached, typically when the change in parameter estimates between iterations falls below a certain threshold.

**Fig. 2:**
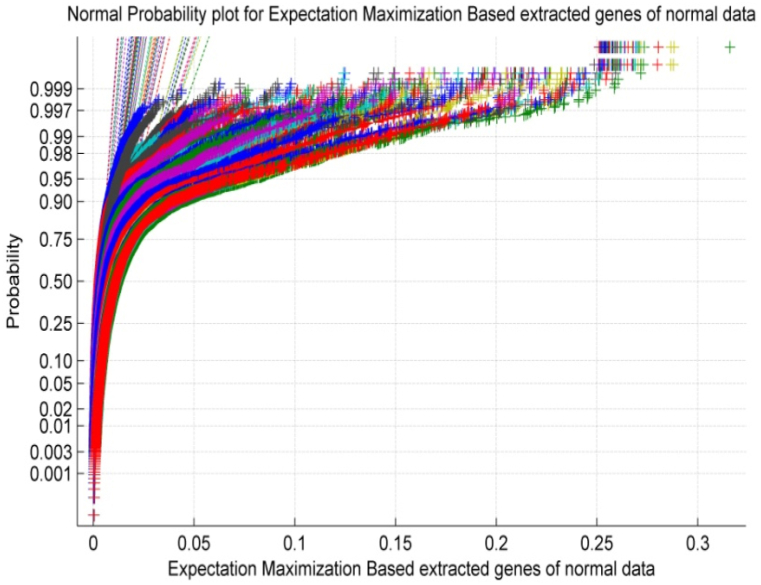
Normal Probability Plot for Extracted features of normal gene data by Expectation Maximization.

As the EM algorithm proceeds, the hidden variables can be used as features for subsequent analysis, such as clustering or classification. The equations for EM can become quite complex, depending on the specific probabilistic model being used. However, the basic idea of alternating between E and M steps remains the same. Using the EM algorithm, 6000 significant genes were extracted per patient from a set of 12,600 prostate microarray gene data points. Fig. 2 exhibits the normal probability plot for Expectation Maximization-based extracted features of Normal microarray gene data. It is observed from Fig. 2 that the normal plot exhibits non-linearity and scattered properties within the normal gene data of the patient. The Expectation Maximization algorithm itself might introduce artifacts that affect the normality of the data.

**Fig. 3:**
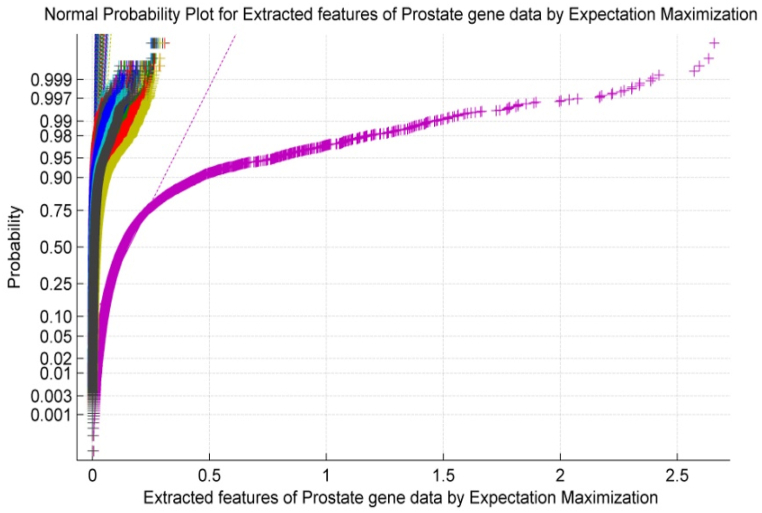
Normal Probability Plot for Extracted features of Prostate gene data by Expectation Maximization.

Fig. 3 exhibits the normal probability plot for Expectation Maximization based extracted features of Prostate microarray gene data. It is observed from Fig. 3 that the normal plot exhibits non-linearity and scattered properties within the Prostate gene data of the patient.

#### 3.2.2 Nonlinear regression

Nonlinear regression is a statistical method used to fit a nonlinear function to a set of data points. It is often used in feature extraction, where the aim is to extract a set of features that best capture the underlying structure of the data [23].

In nonlinear regression, the model takes the form:

(6) m=f(n,θ)+ε

where m is the response variable, n is the predictor variable, θ is a vector of parameters to be estimated, f is a nonlinear function of n and θ, and ε is an error term. The goal is to estimate the values of θ that best fit the data.

Nonlinear regression can employ approximated values of θ features. Steps to explain nonlinear regression:

Choose a nonlinear function f(n, θ) that represents the underlying relationship between the predictor variable x and the response variable m.

Estimate the values of the parameters θ that best fit the data using a nonlinear regression algorithm, such as the Levenberg-Marquardt algorithm.

Use the estimated values of θ as features for subsequent analysis.

The equation for the nonlinear function f(n, θ) will depend on the specific problem being addressed. For example, in a simple case, f(n, θ) might take the form:

(7) $$f\left(n,\theta \right)=\theta_{1}\;*\;\exp\left(-\theta_{2}\;*\;n\right)+\theta_{3}$$

where θ1, θ2, and θ3 are the parameters to be estimated.

The nonlinear regression technique commonly estimates values that minimize the sum of the squared errors between anticipated and actual values of m. After estimating, they can be used as features. The estimated values may be used to classify new data points or cluster them by feature values. Using the NLR algorithm, 6000 significant genes were extracted per patient from a set of 12,600 Prostate microarray gene data points.

**Fig. 4:**
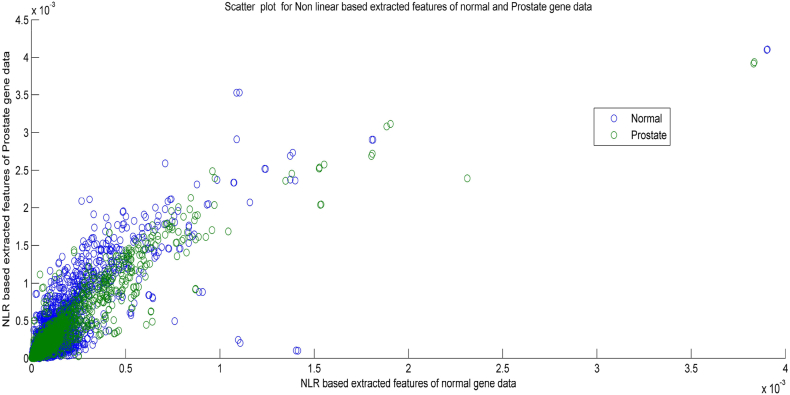
Scatter plot for NLR based extracted features of Normal and Prostate gene data.

Fig. 4 depicts the scatter plot for the extracted features of normal and prostate gene data. It is observed from Fig. 4 that NLR feature extraction method produces non-Gaussian and highly scattered distribution of the variables from the prostate gene data. The data points in the scatter plot don't appear to follow a normal (Gaussian) distribution. This is evident by the lack of a symmetrical bell-shaped curve. The data points in the plot are widely scattered, indicating a high degree of variability in the extracted features between normal and prostate samples.

#### 3.2.3 K means clustering

K-means clustering is a popular unsupervised learning method for microarray gene data feature extraction. K-means clustering divides data points into K user-defined groupings. The centroid of a cluster is its mean data point. In microarray gene data analysis, the data points correspond to the expression levels of different genes across different samples [24]. The goal of K-means clustering in this context is to identify groups of genes that have similar expression patterns across the samples.

The K-means algorithm can be summarized using the following steps:

• Choose a value of K, the number of clusters.
• Initialize the K cluster centroids randomly.
• Assign each gene to the nearest cluster centroid based on the Euclidean distance between the expression levels of the gene and the centroid.
• Recalculate the centroids of each cluster as the mean of the expression levels of the genes assigned to that cluster.
• Repeat steps until convergence, which is typically defined as a small change in the cluster assignments or centroids between iterations.

After K-means convergence, clusters can be used as features for further analysis. For instance, each gene's cluster assignment can be used as a collection of binary characteristics, with 1 if the gene is assigned to that cluster and 0 otherwise. K-means clustering assigns genes to the nearest cluster based on the Euclidean distance between their expression levels and the cluster centroids. After recalculating the centroids as the mean of each cluster's gene expression levels, the process is repeated until convergence is reached. Analysis can use the final cluster assignments. Using the K-means algorithm, 6000 significant genes were extracted per patient from a set of 12,600 prostate microarray gene data points.

#### 3.2.4 Principal Component Analysis (PCA)

Principal Component Analysis (PCA) is a widely used technique for feature extraction and dimensionality reduction in microarray gene data analysis. It can be used to identify the most important patterns of variation in the expression levels of genes across different samples [25].

PCA involves several equations, so here are the main ones:

• Calculate the covariance matrix of the gene expression data:
  o The covariance matrix is a square matrix that shows how each variable (i.e., gene) in the data set is related to every other variable. The equation to calculate the covariance matrix is:

(8) $$Cov\left(x\right)=\frac{1}{n}*\;\left(X-\mu \right)*{\left(X-\mu \right)}^{T}$$

• Where Cov(X) is the covariance matrix, X is the gene expression data matrix (n x m, where n is the number of samples and m is the number of genes), μ is the mean vector of X, and (X - μ) is the centered gene expression data matrix.
• Calculate the eigenvectors and eigenvalues of the covariance matrix:
• The eigenvectors and eigenvalues of the covariance matrix can be calculated using linear algebra. The eigenvectors represent the directions in which the data varies the most, while the eigenvalues represent the amount of variation in each direction. The equation for this step is:

(9) Cov(X)*v=ƛ*v

• Where v is the eigenvector, λ is the corresponding eigenvalue, and Cov(X) is the covariance matrix.
• Sort the eigenvectors by their corresponding eigenvalues in descending order:
• The eigenvectors are sorted in order of importance based on the magnitude of their corresponding eigenvalues.
• Choose the top k eigenvectors that capture the largest amount of variation in the data:
• The top k eigenvectors are chosen based on the amount of variation they capture in the data set. Typically, the top k eigenvectors are chosen to capture a certain percentage of the total variation in the data set.
• Transform the original gene expression data into a new set of features using the selected eigenvectors:
• The original gene expression data can be transformed into a new set of features using the selected eigenvectors. The equation for this step is:

(10) Y=X*V

where Y is the transformed data matrix (n x k, where k is the number of selected eigenvectors), X is the original gene expression data matrix (n x m), and V is the matrix of selected eigenvectors (m x k).

Overall, the goal of PCA is to reduce the dimensionality of the gene expression data by transforming it into a new set of features (i.e., principal components) that capture the most important sources of variation in the data. Using the PCA algorithm, 6000 significant genes were extracted per patient from a set of 12,600 prostate microarray gene data points.

**Fig. 5:**
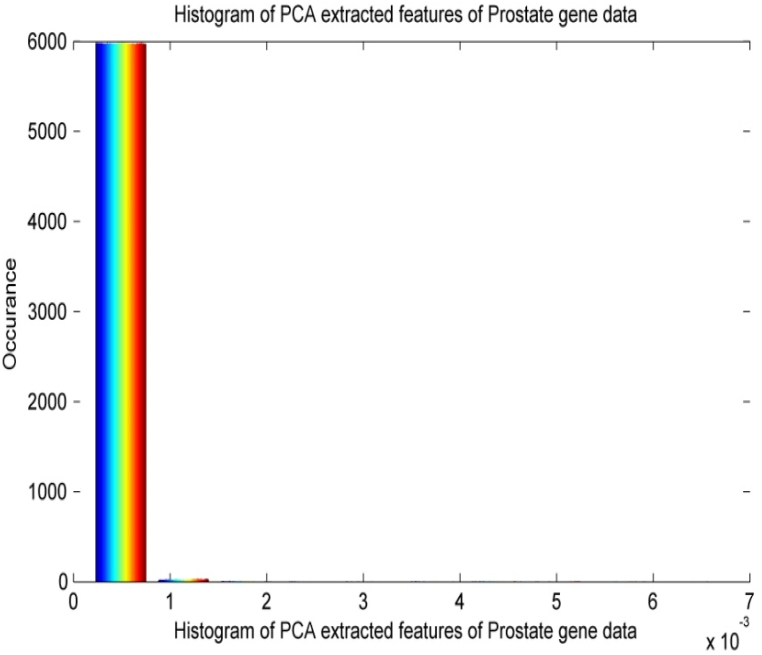
Histogram plot for PCA based extracted features of Prostate gene data.

Fig. 5 shows the histogram plot for PCA-based extracted features of prostate gene data. It is observed from Fig. 5 that the histogram plot shows the presence of non-linearity, scattering, and non-Gaussian properties of the PCA-based feature extraction method for prostate gene data. The distribution is not perfectly symmetrical, suggesting a possible skew towards one side. There are one or more prominent peaks, indicating a concentration of data points at specific feature values.

#### 3.2.5 Discrete Cosine Transform

Discrete Cosine Transform (DCT) is a mathematical technique that can be used for feature extraction in microarray gene data analysis. DCT converts the original gene expression data into a set of coefficients that represent the frequency components of the data. This can help identify the most important patterns of variation in the data [26].

The DCT algorithm can be summarized using the following steps:

1. Divide the gene expression data into smaller segments.
2. Apply the DCT algorithm to each segment to obtain a set of coefficients.
3. Choose the top k coefficients that capture the largest amount of variation in the data, where k is the desired number of features.

For a sequence of N real-valued input values x(n), where 0 ≤ n < N, the DCT can be calculated as follows:

(11) $$X\left(k\right)=\alpha \left(k\right)*\;\sum_{n=0}^{N-1}x\left(n\right)*\;\cos\left[\frac{\pi }{N}*\left(n+0.5\right)*k\right]\;for\;0\leq k\;<\;N$$

where X(k) is the k-th DCT coefficient, and α(k) is a normalization constant given by:

(12) $$\alpha \left(k\right)=\frac{1}{\sqrt{N\;}}\;\text{for}\;k=0$$

(13) $$\alpha \left(k\right)=\sqrt{\frac{2}{N}}\;\text{for}\;k>0$$

The DCT coefficients X(k) represent the frequency content of the input signal, with low-frequency components represented by the lower index k values and high-frequency components represented by the higher index k values. After calculating the DCT coefficients, select the top k depending on magnitude or other criteria. These selected coefficients can be features for further analysis. DCT can identify frequency components in microarray gene expression data to extract useful features. This simplifies data and improves analysis. Using the DCT algorithm, 6000 significant genes were extracted per patient from a set of 12,600 Prostate microarray gene data points.

An ideal normal probability plot would be a straight line. In this figure, the data points deviate from a straight line, particularly curving upwards in the middle. This suggests the extracted features from prostate gene data using DCT might not be normally distributed. The scattered nature of the points reinforces the idea of non-normality. Normally distributed data would tend to fall along a diagonal line.

**Fig. 6:**
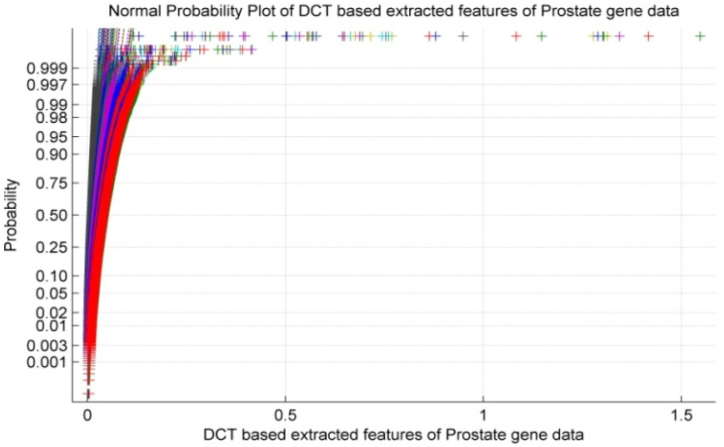
Normal probability plot for extracted features of prostate gene data by discrete cosine transform.

Fig. 6 exhibits the normal probability plot for Discrete Cosine Transform based extracted features of Prostate microarray gene data. It is observed from Fig. 6 that the normal plot exhibits non-linearity and scattered properties within the Prostate gene data of the patient.

#### 3.2.6 Statistical analysis of the feature extraction techniques

Some negative values in features of skewness, kurtosis, and variance indicate the non-linearity present in the extracted features. It is observed from the tables that the statistical parameters depict non-linearity among the patients. This is enriched by the high Skewness and peaked values of kurtosis parameters. The value of Pearson indicates higher variability and implies that these sample features are spread out widely from their mean.

Mean is the average value of the feature across samples. Variance is the measure of how spread out the data points are around the mean. Standard Deviation is the square root of the variance, another measure of spread. Skewness is the measure of the asymmetry of the data distribution (positive for right-skewed, negative for left-skewed). Kurtosis is the measure of the “tailedness” of the data distribution (higher values indicate heavier tails). PCC (Pearson Correlation Coefficient) is the Correlation between the feature and a reference variable (likely another feature). Values closer to 1 indicate strong positive correlation, while values closer to −1 indicate strong negative correlation. Ttest is the statistical test comparing the means of features between normal and cancer data. Values closer to 0 indicate statistically significant differences. p-value<0.01 which indicates whether the T-test p-value is less than 0.01, a common threshold for statistical significance. CCA (Canonical Correlation Analysis) is the correlation between sets of features from normal and cancer data (likely not included in all analyses).

As can be seen in Table 2, the mean and standard deviation of features are not very good across classes, and both skewness and tailed kurtosis show that they are one-sided. The canonical correlations and loadings indicate the degree of correlation between the two sets of variables, but they do not directly measure the degree of separation between classes. If the p-value reported by a *t*-test is less than 0.01, a result is considered statistically significant. If the result has a p-value greater than 0.01, then it cannot be considered significant. From the statistical properties of the features that were extracted using four different methods, as shown in Table 2, even though the CCA values among the groups driven by lower correlation, the histogram and scatter plot show that the features are not Gaussian, have less correlation, overlap, and are skewed, which leads to classification algorithms.

**Table 2:** Average Statistical Features for Dimensionally reduced Prostate Cancer (PCa) and Normal Microarray gene data.

Statistical Features	Dimensionality Reduction Techniques									
	EM		NLR		K Means		PCA		DCT	
	Cancer	Normal	Cancer	Normal	Cancer	Normal	Cancer	Normal	Cancer	Normal
Mean	0.0105	0.0142	0.0001	0.0002	0.7095	0.7076	0.0002	0.0002	0.0250	0.0342
variance	0.0003	0.0014	0.0000	0.0000	0.0006	0.0240	0.0000	0.0000	0.0002	0.0014
std dev	0.0155	0.0215	0.0002	0.0003	0.0235	0.0440	0.0002	0.0001	0.0138	0.0199
Skewness	7.8370	6.6434	8.3360	7.0437	−4.558	−4.37	20.0580	19.4592	14.2057	15.2735
Kurtosis	126.22	83.722	142.277	95.098	39.592	34.571	578.798	553.502	614.013	666.098
PCC	0.7598	0.8557	0.7569	0.8544	0.7626	0.7928	0.8919	0.9258	0.7210	0.8218
Ttest	0.0130	0.4313	0.0159	0.4304	0.0073	0.3970	0.0019	0.4743	0.0001	0.4109
p-value<0.01	0.495	0.334	0.4936	0.334	0.4971	0.3464	0.4992	0.3185	0.4999	0.3413
CCA	0.2847		0.2893		0.2835		0.2818		0.2116	

### 3.3 Feature selection techniques

In this work, meta-heuristic optimization methods like Harmonic Search (HS), Elephant Herd Optimization (EHO) and Firefly are used to choose features from dimensionally reduced data. EHO is a meta-heuristic optimization algorithm based on elephant behavior. Wang et al. [27] shown its use in microarray gene data feature selection. EHO can identify the most dimensionally reduced significant characteristics. The method evaluates a fitness function for each subset of dimensionally reduced features and updates the search process using information from the herd rather than one elephant. HS is a meta-heuristic optimization technique based on cuckoo bird behavior. CS can choose dimensionally reduced, significant features for microarray gene data analysis. CS extracts important features, evaluates a fitness function for each subset of dimensionally reduced relevant features, and updates the search process using cuckoo information. HS, EHO, and FF feature selection increases classification complexity and classifier performance.

#### 3.3.1 Harmonic Search

The improvised style of music served as inspiration for HSA, an evolutionary algorithm. The purpose of this algorithm (HS) is to simulate the improvised performance style of musicians. When improvising in the classical style, performers constantly fine-tune their instruments to ensure a high-quality sound. To initialize the HS algorithm, the Harmony Memory size (SHM), Harmony memory consideration rate (HMCR), Pitch Adjusting Rate (PAR), Bandwidth (BW), Maximum number of Iteration (Maxitr) parameters are considered [28].

• Initialization of Harmony Memory:

In the form of a matrix, all of the decision variables are saved in the memory of the system. The initial HM is obtained for the global optimal solution from a random uniform number of values bounded by kiL and kiv. For the new harmony development considerations of memory, pitch change, and random selection limitations are used to create a new harmony through the solution development phase.

• Harmony memory Updation:

Find the harmony vector fitness function. The new Harmony vector should replace the old one if its fitness function is relatively low. Otherwise, keep the old harmony vector.

• Evaluate the ending criteria:

Repeat steps until maximum number of iterations is achieved Joong Hoon Kim [2016].

#### 3.3.2 Firefly

The goal of optimization is to determine the best possible outcome given a given set of possible options. Back in 2008, Firefly algorithm creator Xin-she Yang [29] made her debut. This program takes its cues from the behavior of fireflies, which attract one another by flashing their lights. There are three rules that explain why fireflies are drawn to each other: (1) despite being sexually dimorphic, fireflies are attracted to the glow of other fireflies of the same gender; (2) the firefly's brightness is a major factor in how appealing it is to potential predators; and (3) fireflies of different genders are not attracted to each other. As long as there is no standout firefly, the others are free to fly wherever they like. (3) The firefly's luminosity reduces with increasing distance because the air medium absorbs some of the light [30].

To enhance the optimization problem, the light intensity I of a firefly at a particular distance ‘k’ can be selected as

(14) I(k)=f(k)

The distance and the brightness of the firefly differ monotonically and exponentially. Based on the inverse square theorem, the brightness of the firefly varies (decreases) with distance.

(15) $$I\propto \frac{1}{r^{2}}$$

The light intensity can be expressed as

(16) $$I=I_{0\;}e^{{-\gamma }^{2}}$$

where γ is the light absorption coefficient, I0 is brightness, and r represents the distance from the source Yang et al., [31]. The brightness or attractiveness of a firefly j, which can be seen in another firefly I, can be calculated as

(17) $$\beta_{i}\left(r\right)=\beta_{i}\left(0\right)\;e^{{-\gamma r}^{2}}$$

Here, β indicates the brightness of the firefly. The Euclidean distance r between the fireflies I and j is given as

(18) $$r=\left\|x_{i}-\;x_{j}\right\|=\sqrt{\sum_{k=1}^{d}{\left(x_{i}-\;x_{j}\right)}^{2}}$$

The positions of the fireflies i and j are indicated as xi, and xj respectively. Assuming, that the firefly j is brighter one, then the movement of the firefly is controlled by its attractiveness, and it's dependent on

(19) $$x_{i}=x_{i}+\left|\beta_{j}\left(r\right)\right|\left(x_{i}-\;x_{j}\right)+\gamma \left(\text{rand}\right)$$

where γ represents the randomization parameter and ‘rnd' denotes any random number obtained from a uniform distribution in the range [1, +1]. The movement of firefly i towards firefly j is regulated by the second term in the above equation. If such an event happens, the third term in the above equation shifts the solution away from the local optimum. The value of the absorption coefficient γ chosen here is 0.1 and j is 25.

#### 3.3.3 Elephant Herding Optimization

Elephant Herding Optimization is a meta-heuristic optimization technique that was created by Wang et al. [32] based on elephant behavior (EHO). When applied to the process of feature selection, it enables the provision of an optimal solution to the associated optimization problems. In the process of feature selection, we choose the aspects of the dependent variable that are likely to be the most illuminating. The EHO consults a herd of elephants in order to find the most effective solution. EHO has the capacity to cope with high-dimensional data and achieve a balance between global and local searches, both of which suggest that it could be useful for feature selection. EHO starts the elephant herd with random feature space positions, elephant positions, and herd activity.

(20) $${{{x_{i}}^{\text{new}}=x}_{i}}^{\text{old}}+\alpha \;\left(\;X_{\text{best}}-\;{X_{i}}^{\text{old}}\right)*r$$

Where ${x_{i}}^{\text{new}}$ and ${X_{i}}^{\text{old}}$ are the old and new positions of the elephant, α∈[0,1] is the control parameter, and r ∈[0,1] is a randomly generated number.

Following is a revised equation for determining the optimal placement.

(21) $$X_{\text{best}}=\beta \;*X_{\text{centre}}$$

(22) $$X_{\text{centre}}=\frac{1}{n}\;*\;\sum_{i=1}^{n}x_{i}$$

Here, $X_{\text{best}}$ is the best position of the elephant with the control parameter as β∈[0,1].

Like the best solution, the worst solution is also accounted for and updated.

(23) $$X_{\text{worst}}=X_{\min}+\left(X_{\max}-X_{\min}+1\;\right)\;*\;\text{rand}$$

Here $X_{\min}$ and $X_{\max}$ represents the maximum and minimum values of the search space.

The selection of optimal parameters for the feature selection algorithms Harmonic Search, Firefly Algorithm, and Elephant Herding Optimization (EHO) is shown in Table 3.

**Table 3:** The selection of Optimal Parameters of the Feature Selection algorithms.

Algorithms	Optimal Parameters of the algorithm
Harmonic Search	HMCR = 0.95, PAR = 0.3, BW = 0.1
Firefly Algorithm	γ = 0.1, $\alpha_{s}\left(0\right)$ = 0.65 initial condition, maximum Iteration of 1000 which ever is earlier.
Elephant HerdingOptimization (EHO)	For a population size of 6000 elephants with a maximum iteration of 1000, and MSE was the selection criteria for α and β through the trial and error method. The α and β are set at critical values of 0.6 and 0.6, respectively.

### 3.4 Classification

The performance of the classifiers was analyzed in this work, with and without feature selection methods. The classifiers used in this research are, SVM (Linear), SVM (Polynomial), SVM (RBF), Random Forest, Decision Tree, QDA, Adaboost and XG boost.

The microarray gene data for prostate cancer exhibits class imbalance, with a significantly higher number of samples from the normal prostate tissue class compared to the prostate cancer tissue class. To address this imbalance, we employed a stratified sampling approach when splitting the data into training and testing sets. Specifically, we used a ratio of 0.1 for training and 0.9 for testing, ensuring that both the training and testing sets maintain the same class distribution as the original dataset. This approach helps prevent the classifiers from being biased towards the majority class and ensures a more robust evaluation of their performance on the imbalanced dataset.

Since the training data for our dataset is limited, we use a technique called k-fold cross-validation to estimate the performance of our machine-learning model. This method, involves dividing the dataset into k equal parts, or “folds.” We then train the model on k-1 folds and test it on the remaining fold. This process is repeated k times, with each fold used once for testing. Afterward, we calculate the average of the performance estimates from each fold to get an overall estimate of the model's performance. Once the model is trained and validated using k-fold cross-validation, we can retrain it on the full dataset and use it to predict new, unseen data. This approach provides a more reliable estimate of the model's performance compared to a simple train-test split, as it utilizes all available data. In our study, we use a k-value of 10-fold.

Training Ratio = *[n*_training_*/ (n*_training_ + *n*_testing_)] × 100%

In this formula:

n _training_ = represents the number of samples in the training set.

n_testing=_represents the number of samples in the testing set.

This formula allows you to calculate the training ratio based on the total number of samples and the number of samples reserved for testing, ensuring a balanced division of the dataset for training and testing purposes.

#### 3.4.1 SVM (Linear)

The SVM linear algorithm is a type of binary classifier that works by finding a hyperplane in the input feature space that separates the data into two classes. The equation for a hyperplane in a two-dimensional space is:

(24) $$w_{1}x_{1}+w_{2}x_{2}+b=0$$

where w1 and w2 are the weights associated with the input features ×1 and ×2, and b is the bias term. The weights and bias terms are learned during the training phase of the SVM algorithm. To classify a new data point (×1, ×2), we plug it into the hyperplane equation to obtain a score:

(25) $$w_{1}x_{1}+w_{2}x_{2}+b=\text{score}$$

If the score is positive, the data point is classified as belonging to one class, and if the score is negative, the data point is classified as belonging to the other class. To visualize the decision boundary, we can plot the input data and the hyperplane in two-dimensional space [33]. The data points are plotted as points with different colors or shapes to represent the two classes, and the hyperplane is plotted as a line. The distance between a data point and the hyperplane is given by:

(26) distance=|score|/‖w‖

where ||w|| is the Euclidean norm of the weight vector w. The SVM algorithm seeks to maximize the distance between the hyperplane and the closest data points in each class, as given by:

(27) margin=2/‖w‖

The optimization problem for the SVM linear algorithm is to minimize the cost function, which penalizes misclassifications and maximizes the margin.

(28) $$\text{minimize}:\frac{1}{2}*\;{\left\|w\right\|}^{2}+C*\text{sum}\;\left(\max\left(0,1-y_{i}*\left(w*x_{i}+b\right)\right)\right)$$

where ||w||^2^ is the squared Euclidean norm of the weight vector w, C is the regularization parameter that controls the trade-off between the margin and the amount of misclassification, y_i_ is the class label of the i^th^ data point, x_i_ is the feature vector of the i^th^ data point, and the sum is over all the training data points. The term max(0, 1 - y_i_ * (w * x_i_ + b)) is called the hinge loss and represents the cost of misclassifying a data point. The SVM algorithm tries to find the values of w and b that minimize the cost function, subject to the constraint that all data points are correctly classified.

#### 3.4.2 SVM (Polynomial)

The polynomial SVM is a type of Support Vector Machine algorithm that can be used for classification tasks. The polynomial SVM finds the best hyperplane that separates the data points into their respective classes using a polynomial kernel function [34]. The decision function for a polynomial SVM can be expressed as:

(29) $$f\left(x\right)=\text{sign}\;\left(\sum_{i=1}^{n}y_{i}\;\alpha_{i}\;\left(1+{\left\langle x_{i},x\right\rangle }^{d}+b\right)\right)$$

where y_i_ is the class label for the ith instance, α_i_ is the Lagrange multiplier corresponding to the ith instance, d is the degree of the polynomial kernel function, x_i_, x is the dot product between the feature vectors of the ith instance and the new instance x, and b is a bias term. The polynomial SVM attempts to maximize the margin between the classes, subject to the constraint that all data points are classified correctly. The optimization problem can be written as:

(30) $$\text{minimize}\frac{1}{2}\;\left(\sum_{i=1}^{n}y_{i}\;{y_{j}\alpha }_{i}\alpha_{j}\;\left(1+{\left\langle x_{i},x_{j}\right\rangle }^{d}\sum_{i=1}^{n}\alpha_{i}\right)\right)$$

(31) $$\text{subject}\;\text{to}\;\sum_{i=1}^{n}{y_{i}\alpha }_{i}=0\;;\;{0\leq \alpha }_{i}\leq C,i=1,\ldots \ldots .,n$$

where C is a hyperparameter that controls the trade-off between maximizing the margin and minimizing the classification error. Once the optimization problem is solved, the decision function can be used to predict the class label of new instances. If f(x) = 0, the new instance is classified as the positive class; otherwise, it is classified as the negative class.

#### 3.4.3 SVM (RBF)

The RBF (Radal Basis Function) SVM is a type of Support Vector Machine algorithm that can be used for classification tasks. The RBF SVM finds the best hyperplane that separates the data points into their respective classes using a radial basis function kernel [35]. The decision function for an RBF SVM can be expressed as:

(32) $$f\left(x\right)=\text{sign}\;\left(\sum_{i=1}^{n}y_{i}\;\alpha_{i}\;K\left\langle x_{i},x\right\rangle +b\right)$$

where yi is the class label for the ith instance, $\alpha_{i}$ is the Lagrange multiplier corresponding to the ith instance, K(x_i_, x) is the RBF kernel function between the ith instance and the new instance x, and b is a bias term. The RBF kernel function takes the form:

(33) $$K\left(x_{i},x_{j}\right)=e^{-{\gamma \left\|x_{i}-\;x_{j}\right\|}^{2}}$$

where γ is a hyperparameter that controls the width of the RBF kernel. The larger the value of gamma, the narrower the kernel and the more localized the decision boundary. The RBF SVM attempts to maximize the margin between the classes, subject to the constraint that all data points are classified correctly. The optimization problem can be written as:

(34) $$\text{minimize}\frac{1}{2}\;\left(\sum_{i=1}^{n}y_{i}\;{y_{j}\alpha }_{i}\alpha_{j}\;\left(K\left\langle x_{i},x_{j}\right\rangle -\sum_{i=1}^{n}\alpha_{i}\right)\right)$$

(35) $$\text{subject}\;\text{to}\;\sum_{i=1}^{n}{y_{i}\alpha }_{i}=0;\;{0\leq \alpha }_{i}\leq C,i=1,\ldots \ldots .,n$$

where C is a hyperparameter that controls the trade-off between maximizing the margin and minimizing the classification error. Once the optimization problem is solved, the decision function can be used to predict the class label of new instances. If f(x) = 0, the new instance is classified as the positive class; otherwise, it is classified as the negative class.

#### 3.4.4 Random forest

Random Forest is a popular machine learning algorithm used for classification tasks. It builds multiple decision trees on randomly selected subsets of data and features, and then combines the outputs of these trees to produce the final prediction [36]. Here is the equation for Random Forest classifier with selection parameters:

Let X be a matrix of input features, and y be a vector of corresponding output labels. The Random Forest algorithm has a number of hyperparameters that can be tuned to optimize its performance.

Some of the important hyperparameters are:

• nestimators: the number of decision trees to be built
• maxdepth: the maximum depth of each decision tree
• maxfeatures: the maximum number of features to be considered at each split

The Random Forest Classifier equation with selection parameters can be written as follows:

• For each decision tree t in range (n_estimators_):
• Randomly sample a subset of data points Dt from the training set with replacement
• Randomly sample a subset of features Ft from the input features without replacement
• Train a decision tree on the subset of data points Dt using the subset of features Ft, with a maximum depth as maxdepth
• For a new input vector x, predict the output label y_pred_ by combining the predictions of all decision trees:

(36) $$y_{pred}=\arg\;\max_{i}\left\{t=1\;n_{estimators}\;\left(1\;if\;t.predict\left(x\right)==i\;else\;0\right)\}\right\}$$

where arg_maxi_ denotes the class i that maximizes the total number of votes across all decision trees. The Random Forest Classifier selects a random subset of data points and features for each decision tree, and then combines the outputs of all decision trees to make the final prediction. The hyperparameters like nestimators, maxdepth, and maxfeatures can be tuned to optimize the performance of the algorithm.

#### 3.4.5 Decision tree

They partition the input space into rectangles or hyper-rectangles, each representing a class or goal value. The decision tree algorithm iteratively splits the input space by feature values until a stopping requirement is reached (e.g., a maximum depth or minimum number of samples per leaf) [37]. Each leaf node represents a class label or target value, whereas each internal node represents a decision rule based on an input feature.

Equations to represent the decision tree algorithm as a classifier:

Given a set of training examples X = {(x_1_, y_1_), (x_2_, y_2_), …, (xn, yn)}, where x_i_ is the ith input feature vector and y_i_ is the corresponding class label:

The decision tree algorithm recursively partitions the input space into regions R_1_, R_2_, …, R_M_ based on the values of input features, such that each region corresponds to a particular class label or target value.

Let R_j_ denote the j^th^ region, and let fj(x) be the decision rule associated with region Rj.

Then, the decision tree can be represented as a set of decision rules in the form of an if-then-else statement:

if fj(x) = true: predict the class label or target value associated with the region.

Rj else: recursively apply the decision tree algorithm to the subset of examples that fall into the region Rj.

The decision rule fj(x) is typically a threshold function of the form:

(37) fj(x) = x_i_≤t_k_

where x_i_ is the value of the i^th^ input feature and t_k_ is a threshold value that determines whether the example falls into region Rj or not.

The threshold value t_k_ is chosen during tree construction to optimize information acquisition or decrease impurity.

Decision trees are pruned to avoid overfitting with training data, and ensemble approaches like random forests or boosting improve their performance and robustness.

#### 3.4.6 Quadrature Discriminant Analysis

Quadrature Discriminant Analysis (QDA) is a classification algorithm used to separate data points into different classes based on a set of features. The QDA model assumes that the data points in each class are normally distributed with their own covariance matrix [38].

Given a set of training data consisting of input vectors x and corresponding output labels y, the QDA classifier can be expressed as follows:

For each class k, compute the mean vector μk and covariance matrix Σ_k_ of the training data points belonging to that class. Then, for a new input vector x, calculate the discriminant function for each class k as:

(38) $$g_{k\left(x\right)}=\ln\left(P\left(y=k\right)\right)\;-\;0.5\;\ln(|\Sigma_{k}|)\;-\;0.5\;{\left(x\;-\;\mu_{k}\right)}^{T}\;\Sigma k^{\left(-1\right)}\;\left(x\;-\;\mu_{k}\right)$$

where |Σk| denotes the determinant of the covariance matrix Σk, and T and (−1) denote the transpose and inverse operations, respectively. Finally, classify the input vector x as belonging to the class with the highest discriminant function value, that is:

(39) $$y=\text{argmaxk}\;\left\{g\;*\;k\left(x\right)\right\}$$

where, argmaxk denotes the class k that maximizes the discriminant function g*k(x). The QDA classifier separates the input space into decision boundaries based on the distribution of the training data for each class and then assigns new data points to the class with the highest discriminant function value.

#### 3.4.7 Adaboost

AdaBoost is a popular machine learning algorithm used for classification tasks. It works by combining multiple weak learners (classifiers that perform slightly better than random guessing) into a strong learner that can make accurate predictions [39]. Here is the equation for the AdaBoost classifier with selection parameters:

Let X be a matrix of input features, and y be a vector of corresponding output labels. The AdaBoost algorithm has a number of hyperparameters that can be tuned to optimize its performance.

Some of the important hyperparameters are:

• n_estimators_: the maximum number of weak learners to be used
• learning_rate_: the contribution of each weak learner to the final prediction
• base_estimator_: the type of weak learner to be used (e.g., decision tree)

The AdaBoost classifier equation with selection parameters can be written as follows:

• Initialize the weights wi for each training data point (i.e., wi = 1/N, where N is the number of training data points)
• For each weak learner t in range(n_estimators_):
  a Train a weak learner on the training data using the current weights wi
  b Compute the weighted error εt of the weak learner on the training data
  c Compute the contribution αt of the weak learner to the final prediction:

(40) αt=learning_rate*ln((1−εt)/εt)

d. Update the weights w for the next iteration:

wi = wi * exp(-αt * yi * t.predict(Xi)) / Zt

where Zt is a normalization factor that ensures the weights sum up to 1.

For a new input vector x, predict the output label ypred by combining the predictions of all weak learners:

ypred = sign(sum_t = 1n_estimators_ α_t_ *t.predict(x))

where sign() returns −1 if the argument is negative, 0 if it is zero, and 1 if it is positive. In summary, the AdaBoost Classifier iteratively trains multiple weak learners on the training data and then combines their predictions to make the final prediction. The hyperparameters like nestimators, learning_rate, and base_estimator can be tuned to optimize the performance of the algorithm.

#### 3.4.8 XG boost

XGBoost is an algorithm for supervised learning, which means it is used for classification and regression problems [40]. The basic equation for XGBoost as a classifier can be represented as follows:

(41) $$\hat{y_{i}\;}=\text{softmax}\;\left(\sum_{k=1}^{K}f_{k}\left(x_{i}\right)\right)$$

where hat{yi} is the predicted class label for the ith instance, K is the total number of trees in the ensemble, fk is the kth decision tree, and xi is the feature vector for the ith instance. In the case of binary classification, the predicted probability for the positive class can be calculated as follows:

(42) $$\hat{p_{i}}=\frac{1}{1+e\;\sum_{k=1}^{K}f_{k}\left(x_{i}\right)}$$

where hat{pi} is the predicted probability for the positive class of the ith instance. Each decision tree in the XGBoost ensemble is built iteratively, with each new tree attempting to correct the errors made by the previous trees. The objective function for training the XGBoost model is defined as the sum of a loss function L and a regularization term R:

(43) $$\text{obj}\;\left(\Theta \right)=\sum_{i=1}^{n}L\left(y_{i},\hat{y_{i}}\right)+\sum_{i=1}^{K}\Omega \;\left(f_{k}\right)$$

where Θ represents the set of model parameters, yi is the true class label for the ith instance, and Omega(fk) is the regularization term for the kth decision tree. The XGBoost algorithm minimizes this objective function using gradient boosting, which involves iteratively adding decision trees to the ensemble that minimize the objective function. Then, a majority vote of the predictions from all of the ensemble's trees determines the final prediction.

The classifiers are trained with a 10-fold training and testing method along with a MSE value of 10^−05^ or a maximum iteration of 1000, whichever is earlier. as the stopping criteria. The following Table 4 shows the selection of optimal parameters for the classifiers.

**Table 4:** The selection of Optimal Parameters of the classifiers.

Classifier	Optimal parameters of the classifiers
SVM (Linear)	Regularization parameter C: 10, Criterion: MSE
SVM (Polynomial)	Degree of the polynomial kernel function: 3, Criterion: MSE
SVM (RBF)	C: The regularization parameter 0.5 γ = 0.1 Criterion: MSE
Random forest	n_estimators_ = 100, maxdepth = 5, and maxfeatures = 10, Criterion: MSE
Decision tree	Max depth: 10, min samples: 1 Criterion: MSE
QDA	The regularization parameter 0.5 Criterion: MSE
Adaboost	n_estimators_ = 100, learning_rate = 0.1, and iterations – 100, Criterion: MSE
XG boost	n_estimators_ = 100, learning_rate = 0.01, Criterion: MSE

## 4 Results and discussion

The metrics such as accuracy, precision, Gmean, F1 score, Kappa, MCC, error rate, FM, Jaccard metric, and CSI are commonly used in binary classification to evaluate the performance of classifiers. These metrics provide insights into different aspects of the classifier's performance, such as its ability to correctly classify positive and negative instances, its overall accuracy, and its balance between precision and recall. By considering these metrics, we can assess the effectiveness of the classifier in distinguishing between the two classes and make informed decisions about its performance.

Accuracy measures overall correct predictions, precision focuses on correct positive predictions, and Gmean balances sensitivity and specificity. The F1 score combines precision and recall, Kappa assesses agreement beyond chance, and MCC indicates the quality of predictions. Error rate quantifies incorrect predictions, FM measures cluster similarity, Jaccard metric assesses set similarity, and CSI emphasizes correct predictions for both classes. These metrics collectively provide a comprehensive view of classifier performance, considering various aspects of accuracy and reliability in classification.

The output of a classifier's prediction for a given class may result in one of four potential outcomes, based on the classification. These outcomes include True Positive (TP), which identifies a normal class accurately; True Negative (TN), which identifies an abnormal class accurately; False Positive (FP), which misidentifies an abnormal class as normal; and False Negative (FN), which misidentifies a normal class as abnormal. These outcomes are taken into account when evaluating classification performance using the metrics mentioned below.

From Table 5, it can be observed that different classifiers exhibit varying performance across the different feature extraction techniques for prostate cancer classification using microarray gene data. **Expectation Maximization (EM) based Features with** SVM (Linear) shows the lowest performance with an accuracy of 55.88 % and a high error rate of 44.12 %. Precision is also low at 62.32 %, indicating a high false positive rate. Other metrics like MCC, Gmean, and Kappa are also low, suggesting poor overall performance. In the **Non-Linear Regression (NLR) based Features**, SVM (RBF) performs significantly better compared to SVM (Linear) with an accuracy of 75.74 % and a low error rate of 24.26 %. XGboost achieves the highest accuracy of 78.68 % among all classifiers with NLR features. For the **K Means Algorithm based Features**, SVM (RBF) shows the highest performance with an accuracy of 76.47 % and a low error rate of 23.53 %. Decision tree and QDA also perform reasonably well with accuracies above 72 %. In the **Principal Component Analysis (PCA) based Features**, SVM (RBF) achieves an accuracy of 72.06 % with PCA features, which is lower than its performance with NLR and K Means features. XGboost exhibits the highest accuracy of 71.32 % among all classifiers with PCA features. In the **Discrete Cosine Transform (DCT) based Features**, SVM (RBF) shows the highest performance with an accuracy of 79.41 % and a low error rate of 20.59 %. XGboost achieves the highest accuracy of 86.03 % among all classifiers with DCT features, indicating the effectiveness of DCT in feature extraction for prostate cancer classification.

**Table 5:** Classifiers' Performance Metrics for Prostate data for different feature extraction methods.

Classifiers	Performance Measures									
	Accuracy	Precision	F1Score	MCC	FM	Error Rate	Jaccard Metric	CSI	Gmean	Kappa
**Expectation Maximization**										
SVM (Linear)	55.88	62.32	58.90	0.12	0.59	44.12	41.75	18.16	55.40	0.12
SVM (Polynomial)	57.35	63.77	60.27	0.15	0.60	42.65	43.14	20.91	56.89	0.15
SVM (RBF)	69.12	80.70	68.66	0.41	0.69	30.88	52.27	40.44	70.02	0.40
Random forest	62.50	68.57	65.31	0.25	0.65	37.50	48.48	30.91	62.00	0.25
Decision tree	62.50	67.11	66.67	0.24	0.67	37.50	50.00	33.34	61.67	0.24
QDA	65.44	72.06	67.59	0.31	0.68	34.56	51.04	35.70	65.11	0.31
Adaboost	69.12	73.97	72.00	0.38	0.72	30.88	56.25	44.10	68.53	0.38
XG boost	72.79	77.78	75.17	0.45	0.75	27.21	60.22	50.51	72.29	0.45
**Non Linear Regression**										
SVM (Linear)	58.09	66.67	58.39	0.18	0.59	41.91	41.24	18.61	58.49	0.17
SVM (Polynomial)	68.38	72.37	71.90	0.36	0.72	31.62	56.12	43.80	67.70	0.36
SVM (RBF)	75.74	82.35	77.24	0.52	0.77	24.26	62.92	55.08	75.45	0.51
Random forest	72.06	83.05	72.06	0.47	0.73	27.94	56.32	46.69	72.70	0.45
Decision tree	73.53	83.61	73.91	0.49	0.74	26.47	58.62	49.84	73.91	0.48
QDA	73.53	80.60	75.00	0.48	0.75	26.47	60.00	50.73	73.30	0.47
Adaboost	74.26	76.92	77.42	0.48	0.77	25.74	63.16	54.85	73.74	0.48
XG boost	78.68	82.43	80.79	0.57	0.81	21.32	67.78	61.65	78.20	0.57
**K means algorithm**										
SVM (Linear)	59.56	66.67	61.54	0.20	0.62	40.44	44.44	23.81	59.36	0.19
SVM (Polynomial)	59.56	65.71	62.59	0.19	0.63	40.44	45.54	25.45	59.03	0.19
SVM (RBF)	76.47	84.62	77.46	0.54	0.78	23.53	63.22	56.04	76.42	0.53
Random forest	72.79	82.26	73.38	0.47	0.74	27.21	57.95	48.49	73.05	0.46
Decision tree	57.35	63.77	60.27	0.15	0.60	42.65	43.14	20.91	56.89	0.15
QDA	72.06	76.71	74.67	0.44	0.75	27.94	59.57	49.44	71.51	0.44
Adaboost	72.79	76.32	75.82	0.45	0.76	27.21	61.05	51.64	72.21	0.45
XG boost	83.82	87.67	85.33	0.67	0.85	16.18	74.42	70.79	83.41	0.67
**Principal Component Analysis**										
SVM (Linear)	58.82	65.67	61.11	0.18	0.61	41.18	44.00	22.81	58.54	0.18
SVM (Polynomial)	57.35	63.77	60.27	0.15	0.60	42.65	43.14	20.91	56.89	0.15
SVM (RBF)	72.06	85.45	71.21	0.48	0.72	27.94	55.29	46.49	73.35	0.45
Random forest	66.91	72.86	69.39	0.34	0.69	33.09	53.13	39.09	66.45	0.34
Decision tree	66.91	72.22	69.80	0.33	0.70	33.09	53.61	39.75	66.34	0.33
QDA	72.79	80.30	74.13	0.46	0.74	27.21	58.89	49.13	72.64	0.46
Adaboost	68.38	75.00	70.34	0.37	0.70	31.62	54.26	41.23	68.06	0.37
XG boost	71.32	76.39	73.83	0.42	0.74	28.68	58.51	47.82	70.80	0.42
**Discrete Cosine Transform**										
SVM (Linear)	59.56	66.18	62.07	0.19	0.62	40.44	45.00	24.62	59.19	0.19
SVM (Polynomial)	62.50	68.57	65.31	0.25	0.65	37.50	48.48	30.91	62.00	0.25
SVM (RBF)	79.41	86.57	80.56	0.60	0.81	20.59	67.44	61.89	79.20	0.59
Random forest	75.00	75.90	78.75	0.49	0.79	25.00	64.95	57.72	74.74	0.48
Decision tree	83.09	86.49	84.77	0.66	0.85	16.91	73.56	69.60	82.68	0.66
QDA	64.71	70.42	67.57	0.29	0.68	35.29	51.02	35.36	64.16	0.29
Adaboost	72.79	78.57	74.83	0.46	0.75	27.21	59.78	50.00	72.37	0.45
XG boost	86.03	90.28	87.25	0.72	0.87	13.97	77.38	74.69	85.65	0.72

In conclusion, the choice of feature extraction technique significantly impacts the performance of classifiers for prostate cancer classification using microarray gene data. DCT features appear to be the most effective based on the results, particularly when used with the XGboost classifier. However, further research and experimentation may be needed to validate these findings and explore other potential feature extraction methods and classifiers.

Table 6 presents the performance metrics of various classifiers for prostate cancer classification using different feature extraction methods, with Harmonic Search employed as the feature selection technique. For the Expectation Maximization (EM) based features, SVM (RBF) stands out as the top performer with an accuracy of 84.56 % and a high precision of 90.00 %. This indicates that SVM (RBF) can effectively distinguish between the two classes in the dataset. Non-Linear Regression (NLR) based features also show strong performance, especially with the QDA classifier, which achieves an accuracy of 86.77 % and a precision of 89.33 %. This suggests that the NLR features contain valuable information for classification. K Means algorithm based features, however, do not perform as well, with most classifiers showing lower accuracies and precisions compared to other feature extraction methods. Principal Component Analysis (PCA) based features yield mixed results, with SVM (RBF) achieving an accuracy of 74.27 % and an F1 score of 74.45 %, indicating moderate performance. The Discrete Cosine Transform (DCT) based features perform reasonably well, with SVM (RBF) achieving an accuracy of 80.88 % and a precision of 84.00 %.

**Table 6:** Classifiers' Performance Metrics for Prostate data for different feature extraction methods with Harmonic Search as a feature selection.

Classifiers	Performance Measures									
	Accuracy	Precision	F1Score	MCC	FM	Error Rate	Jaccard Metric	CSI	Gmean	Kappa
**Expectation Maximization**										
SVM (Linear)	63.24	68.00	67.11	0.26	0.67	36.77	50.50	34.23	62.46	0.26
SVM (Polynomial)	70.59	77.61	72.22	0.42	0.72	29.41	56.52	45.14	70.35	0.41
SVM (RBF)	84.56	90.00	85.71	0.69	0.86	15.44	75.00	71.82	84.21	0.69
Random forest	82.35	85.33	84.21	0.64	0.84	17.65	72.73	68.45	81.94	0.64
Decision tree	72.79	76.32	75.82	0.45	0.76	27.21	61.05	51.64	72.21	0.45
QDA	84.56	87.84	86.09	0.69	0.86	15.44	75.58	72.25	84.17	0.69
Adaboost	79.41	83.56	81.33	0.59	0.81	20.59	68.54	62.78	78.96	0.58
XG boost	81.62	86.11	83.22	0.63	0.83	18.38	71.26	66.63	81.20	0.63
**Non Linear Regression**										
SVM (Linear)	58.09	64.71	60.69	0.16	0.61	41.91	43.56	21.85	57.71	0.16
SVM (Polynomial)	58.82	64.79	62.16	0.17	0.62	41.18	45.10	24.53	58.22	0.17
SVM (RBF)	83.82	85.71	85.71	0.67	0.86	16.18	75.00	71.43	83.51	0.67
Random forest	72.79	83.33	72.99	0.48	0.74	27.21	57.47	48.27	73.30	0.46
Decision tree	73.53	81.54	74.65	0.48	0.75	26.47	59.55	50.37	73.47	0.47
QDA	86.77	89.33	88.16	0.73	0.88	13.24	78.82	76.35	86.42	0.73
Adaboost	80.15	83.78	82.12	0.60	0.82	19.85	69.66	64.30	79.70	0.60
XG boost	83.82	86.67	85.53	0.67	0.86	16.18	74.71	71.08	83.44	0.67
**K means algorithm**										
SVM (Linear)	54.41	60.87	57.53	0.09	0.58	45.59	40.39	15.42	53.92	0.09
SVM (Polynomial)	58.82	64.79	62.16	0.17	0.62	41.18	45.10	24.53	58.22	0.17
SVM (RBF)	72.06	85.46	71.21	0.48	0.72	27.94	55.29	46.49	73.35	0.46
Random forest	71.32	81.67	71.53	0.45	0.72	28.68	55.68	45.30	71.82	0.44
Decision tree	67.65	73.24	70.27	0.35	0.70	32.35	54.17	40.77	67.14	0.35
QDA	72.06	76.71	74.67	0.44	0.75	27.94	59.57	49.44	71.51	0.44
Adaboost	80.88	85.92	82.43	0.62	0.83	19.12	70.12	65.14	80.48	0.62
XG boost	84.56	87.84	86.09	0.69	0.86	15.44	75.58	72.25	84.17	0.69
**Principal Component Analysis**										
SVM (Linear)	55.88	62.32	58.90	0.12	0.59	44.12	41.75	18.16	55.40	0.12
SVM (Polynomial)	57.35	63.77	60.27	0.15	0.60	42.65	43.14	20.91	56.89	0.15
SVM (RBF)	74.27	85.00	74.45	0.51	0.75	25.74	59.30	51.23	74.78	0.49
Random forest	79.41	83.56	81.33	0.59	0.81	20.59	68.54	62.78	78.96	0.58
Decision tree	73.53	82.54	74.29	0.49	0.75	26.47	59.09	50.07	73.67	0.48
QDA	75.00	76.54	78.48	0.49	0.79	25.00	64.58	57.06	74.61	0.49
Adaboost	76.47	77.78	79.75	0.52	0.80	23.53	66.32	59.60	76.14	0.52
XG boost	72.79	78.57	74.83	0.46	0.75	27.21	59.78	50.00	72.38	0.45
**Discrete Cosine transform**										
SVM (Linear)	55.88	61.97	59.46	0.11	0.60	44.12	42.31	19.12	55.24	0.11
SVM (Polynomial)	58.09	63.51	62.25	0.15	0.62	41.91	45.19	24.55	57.26	0.15
SVM (RBF)	80.88	84.00	82.90	0.61	0.83	19.12	70.79	65.82	80.45	0.61
Random forest	73.53	78.87	75.68	0.47	0.76	26.47	60.87	51.60	73.07	0.47
Decision tree	78.68	82.43	80.80	0.57	0.81	21.32	67.78	61.65	78.21	0.57
QDA	84.56	86.84	86.28	0.69	0.86	15.44	75.86	72.56	84.22	0.69
Adaboost	74.27	75.00	78.26	0.47	0.78	25.74	64.29	56.82	74.03	0.47
XG boost	77.21	79.49	80.00	0.54	0.80	22.79	66.67	60.01	76.77	0.54

Finally, the choice of feature extraction method has a significant impact on the performance of classifiers for prostate cancer classification. Further research could explore additional feature selection techniques and classifiers to improve classification accuracy and reliability.

Table 7 displays the performance analysis of the eight classifiers in terms of parameters such as accuracy, precision, Gmean, F1 score, Kappa, MCC, error rate, FM, Jaccard metric and CSI values for five feature extraction techniques: EM, NLR, K-means, PCA, and DCT, with the features selected by the Firefly algorithm. From Table 7, it is also examined that the SVM (Linear) classifier is a low-performing one with an accuracy of 56.62 %, an F1 score of 59.86 % and a high error rate of 43.38 % for the extracted features by the K-Means algorithm. The SVM (Linear) classifier is also showing a low value of MCC 0.13 and a Kappa value of 0.13. The lack of repeatability is demonstrated in the SVM (Linear) classifier with a low precision of 62.86 % for the extracted features by the K-Means algorithm. The XGBoost classifier is a high-performing one for the Non Linear Regression based feature extraction method. The XGboost classifier reached a high accuracy of 88.97 %, with a low error rate of 11.03 % and a good F1 score of 90.32 %, along with a Gmean of 88.83 %. The higher MCC and Kappa values of 0.78 and 0.78 are achieved by the XGboost classifier. In summary, SVM (RBF), Adaboost, and XG boost classifiers demonstrate better performance compared to SVM (Linear) and other classifiers across different feature extraction methods with the Firefly algorithm as a feature selection technique. These classifiers show higher accuracy, precision, F1 scores, MCC, Gmean, and Kappa values, indicating their suitability for the prostate cancer classification task.

**Table 7:** Classifiers' Performance Metrics for Prostate data for different feature extraction methods with Firefly algorithm as a feature selection.

Classifiers	Performance Measures									
	Accuracy	Precision	F1Score	MCC	FM	Error Rate	Jaccard Metric	CSI	Gmean	Kappa
**Expectation Maximization**										
SVM (Linear)	60.29	67.16	62.50	0.21	0.63	39.71	45.46	25.61	60.01	0.21
SVM (Polynomial)	72.79	85.71	72.18	0.49	0.73	27.21	56.47	48.05	73.92	0.47
SVM (RBF)	72.79	84.48	72.59	0.49	0.73	27.21	56.98	48.12	73.59	0.47
Random forest	72.79	83.33	72.99	0.48	0.74	27.21	57.47	48.27	73.30	0.46
Decision tree	72.79	78.57	74.83	0.46	0.75	27.21	59.78	50.00	72.38	0.45
QDA	75.00	75.90	78.75	0.49	0.79	25.00	64.95	57.72	74.74	0.49
Adaboost	86.03	89.19	87.42	0.72	0.87	13.97	77.65	74.90	85.65	0.72
XG boost	86.03	90.28	87.25	0.72	0.87	13.97	77.38	74.69	85.65	0.72
**Non Linear Regression**										
SVM (Linear)	66.91	73.53	68.97	0.34	0.69	33.09	52.63	38.46	66.58	0.34
SVM (Polynomial)	73.53	82.54	74.29	0.49	0.75	26.47	59.09	50.07	73.67	0.48
SVM (RBF)	78.68	88.71	79.14	0.59	0.80	21.32	65.48	60.14	78.95	0.58
Random forest	72.79	76.32	75.82	0.45	0.76	27.21	61.05	51.64	72.21	0.45
Decision tree	78.68	83.33	80.54	0.57	0.81	21.32	67.42	61.26	78.23	0.57
QDA	82.35	86.30	84.00	0.65	0.84	17.65	72.41	68.12	81.93	0.64
Adaboost	85.29	89.04	86.67	0.70	0.87	14.71	76.47	73.46	84.90	0.70
XG boost	88.97	89.74	90.32	0.78	0.90	11.03	82.35	80.65	88.83	0.78
**K means algorithm**										
SVM (Linear)	56.62	62.86	59.86	0.13	0.60	43.38	42.72	20.00	56.06	0.13
SVM (Polynomial)	57.35	63.77	60.27	0.15	0.60	42.65	43.14	20.91	56.89	0.15
SVM (RBF)	73.53	88.68	72.31	0.52	0.74	26.47	56.63	49.72	75.25	0.49
Random forest	83.82	85.71	85.71	0.67	0.86	16.18	75.00	71.43	83.51	0.67
Decision tree	73.53	76.62	76.62	0.46	0.77	26.47	62.11	53.25	72.97	0.46
QDA	75.74	76.83	79.25	0.50	0.79	24.27	65.63	58.65	75.44	0.50
Adaboost	83.82	87.67	85.33	0.67	0.85	16.18	74.42	70.79	83.42	0.67
XG boost	86.03	88.16	87.58	0.72	0.88	13.97	77.91	75.17	85.71	0.72
**Principal Component Analysis**										
SVM (Linear)	57.35	64.62	59.16	0.15	0.59	42.65	42.00	19.16	57.24	0.15
SVM (Polynomial)	58.82	66.67	60.00	0.19	0.60	41.18	42.86	21.21	58.91	0.18
SVM (RBF)	85.29	92.54	86.11	0.71	0.86	14.71	75.61	73.06	85.10	0.71
Random forest	60.29	67.69	61.97	0.21	0.62	39.71	44.90	24.84	60.19	0.21
Decision tree	72.06	85.46	71.21	0.48	0.72	27.94	55.29	46.49	73.35	0.46
QDA	73.53	82.54	74.29	0.49	0.75	26.47	59.09	50.07	73.67	0.48
Adaboost	73.53	83.61	73.91	0.49	0.74	26.47	58.62	49.84	73.91	0.48
XG boost	81.62	80.95	84.47	0.62	0.85	18.38	73.12	69.26	81.82	0.62
**Discrete Cosine Transform**										
SVM (Linear)	72.79	80.30	74.13	0.46	0.74	27.21	58.89	49.13	72.64	0.46
SVM (Polynomial)	72.06	77.47	74.32	0.44	0.74	27.94	59.14	48.89	71.59	0.44
SVM (RBF)	74.27	78.38	76.82	0.48	0.77	25.74	62.37	53.70	73.73	0.48
Random forest	75.00	76.54	78.48	0.49	0.79	25.00	64.58	57.06	74.61	0.49
Decision tree	75.00	75.90	78.75	0.49	0.79	25.00	64.95	57.72	74.74	0.49
QDA	83.82	86.67	85.53	0.67	0.86	16.18	74.71	71.08	83.44	0.67
Adaboost	85.29	88.00	86.84	0.70	0.87	14.71	76.74	73.71	84.93	0.70
XG boost	86.03	89.19	87.42	0.72	0.87	13.97	77.65	74.90	85.65	0.72

In Table 8, the classifiers' performance metrics for prostate cancer classification using different feature extraction methods with Elephant Herding Optimization (EHO) as a feature selection technique are presented. In **SVM (RBF)** classifier consistently performs well across all feature extraction methods, with accuracy ranging from 86.03 % to 94.85 %. Precision, F1 scores, MCC, Gmean, and Kappa values are also high, indicating its robustness in classifying prostate cancer using the selected features. For the **Adaboost** and **XG boost** classifiers also demonstrate high performance, with accuracy ranging from 85.29 % to 91.91 % and 88.24 %–94.12 %, respectively. They exhibit high precision, F1 scores, MCC, Gmean, and Kappa values, suggesting their effectiveness in prostate cancer classification with EHO-selected features. The Quadratic Discriminant Analysis classifier shows varying performance across feature extraction methods, with accuracy ranging from 77.21 % to 90.44 %. While generally performing well, it shows slightly lower performance compared to SVM (RBF), Adaboost, and XG boost in most cases. **Random forest** and **Decision tree** classifiers exhibit moderate to high performance, with accuracy ranging from 73.53 % to 84.56 % and 74.27 %–88.24 %, respectively. They show good precision, F1 scores, MCC, Gmean, and Kappa values, indicating their utility in prostate cancer classification with EHO-selected features. **Non-linear Regression** and **Principal Component Analysis** classifiers show moderate performance, with accuracy ranging from 57.35 % to 86.77 % and 63.97 %–91.18 %, respectively. While they perform well in some cases, they exhibit lower performance compared to SVM (RBF), Adaboost, and XG boost in many instances. **K means algorithm** classifier shows mixed performance, with accuracy ranging from 58.09 % to 90.44 %. It performs well in some cases but shows lower performance compared to SVM (RBF), Adaboost, and XG boost in others.

**Table 8:** Classifiers' Performance Metrics for Prostate data for different feature extraction methods with Elephant Herding Optimization feature selection.

Classifiers	Performance Measures									
	Accuracy	Precision	F1Score	MCC	FM	Error Rate	Jaccard Metric	CSI	Gmean	Kappa
**Expectation Maximization**										
SVM (Linear)	56.62	62.86	59.86	0.13	0.60	43.38	42.72	20.00	56.06	0.13
SVM (Polynomial)	61.77	67.61	64.87	0.23	0.65	38.24	48.00	29.94	61.19	0.23
SVM (RBF)	86.03	88.16	87.58	0.72	0.88	13.97	77.91	75.17	85.71	0.72
Random forest	73.53	76.62	76.62	0.46	0.77	26.47	62.11	53.25	72.97	0.46
Decision tree	74.27	76.25	77.71	0.47	0.78	25.74	63.54	55.47	73.80	0.47
QDA	83.82	87.67	85.33	0.67	0.85	16.18	74.42	70.79	83.42	0.67
Adaboost	85.29	89.04	86.67	0.70	0.87	14.71	76.47	73.46	84.90	0.70
XG boost	88.24	90.67	89.47	0.76	0.90	11.77	80.95	78.98	87.92	0.76
**Non Linear Regression**										
SVM (Linear)	57.35	63.77	60.27	0.15	0.60	42.65	43.14	20.91	56.89	0.15
SVM (Polynomial)	72.79	79.41	74.48	0.46	0.75	27.21	59.34	49.54	72.49	0.46
SVM (RBF)	86.77	87.34	88.46	0.73	0.89	13.24	79.31	76.95	86.65	0.73
Random forest	83.09	86.49	84.77	0.66	0.85	16.91	73.56	69.60	82.68	0.66
Decision tree	84.56	86.84	86.28	0.69	0.86	15.44	75.86	72.56	84.22	0.69
QDA	77.21	77.38	80.75	0.53	0.81	22.79	67.71	61.80	77.15	0.53
Adaboost	75.00	76.54	78.48	0.49	0.79	25.00	64.58	57.06	74.61	0.49
XG boost	88.24	90.67	89.47	0.76	0.90	11.77	80.95	78.98	87.92	0.76
**K means algorithm**										
SVM (Linear)	63.97	70.59	66.21	0.28	0.66	36.03	49.49	32.93	63.63	0.28
SVM (Polynomial)	58.09	64.29	61.22	0.16	0.61	41.91	44.12	22.73	57.55	0.16
SVM (RBF)	90.44	93.24	91.39	0.81	0.91	9.56	84.15	82.85	90.12	0.81
Random forest	75.00	81.16	76.71	0.50	0.77	25.00	62.22	53.89	74.65	0.50
Decision tree	75.00	76.54	78.48	0.49	0.79	25.00	64.58	57.06	74.61	0.49
QDA	75.00	75.29	79.01	0.49	0.79	25.00	65.31	58.41	74.90	0.48
Adaboost	72.79	76.32	75.82	0.45	0.76	27.21	61.05	51.64	72.21	0.45
XG boost	81.62	83.33	83.87	0.63	0.84	18.38	72.22	67.75	81.30	0.63
**Principal Component Analysis**										
SVM (Linear)	72.06	78.26	73.97	0.44	0.74	27.94	58.70	48.39	71.69	0.44
SVM (Polynomial)	63.97	68.92	67.55	0.27	0.68	36.03	51.00	35.15	63.26	0.27
SVM (RBF)	91.18	93.33	92.11	0.82	0.92	8.82	85.37	84.24	90.90	0.82
Random forest	84.56	87.84	86.09	0.69	0.86	15.44	75.58	72.25	84.17	0.69
Decision tree	84.56	87.84	86.09	0.69	0.86	15.44	75.58	72.25	84.17	0.69
QDA	88.24	90.67	89.47	0.76	0.90	11.77	80.95	78.98	87.92	0.76
Adaboost	89.71	92.00	90.79	0.79	0.91	10.29	83.13	81.61	89.41	0.79
XG boost	91.18	93.33	92.11	0.82	0.92	8.82	85.37	84.24	90.90	0.82
**Discrete Cosine Transform**										
SVM (Linear)	63.24	68.49	66.67	0.26	0.67	36.77	50.00	33.43	62.56	0.26
SVM (Polynomial)	72.06	80.00	73.24	0.45	0.74	27.94	57.78	47.53	71.99	0.44
SVM (RBF)	94.85	97.30	95.36	0.90	0.95	5.15	91.14	90.80	94.58	0.90
Random forest	80.88	84.00	82.90	0.61	0.83	19.12	70.79	65.82	80.45	0.61
Decision tree	88.24	91.78	89.33	0.76	0.89	11.77	80.72	78.79	87.87	0.76
QDA	90.44	93.24	91.39	0.81	0.91	9.56	84.15	82.85	90.12	0.81
Adaboost	91.91	94.60	92.72	0.84	0.93	8.09	86.42	85.50	91.61	0.84
XG boost	94.12	98.59	94.60	0.89	0.95	5.88	89.74	89.50	93.79	0.88

In conclusion, SVM (RBF), Adaboost, and XG boost classifiers demonstrate superior performance in classifying prostate cancer using EHO-selected features, exhibiting high accuracy, precision, F1 scores, MCC, Gmean, and Kappa values. These classifiers are recommended for further investigation and potential application in clinical practice for prostate cancer diagnosis and treatment planning.

Table 9 presents the best methodology for different approaches using different classifiers with average accuracy results. The table displays the best accuracy results with and without feature selection techniques individually, as well as the overall average accuracy performance for all methodologies. The best methodology is DCT (Discrete Cosine Transform) combined with XGboost, achieving an average accuracy of 86.03 % Without Feature Selection. **Feature selection by Harmonic Search** gives NLR (Non Linear Regression) combined with QDA (Quadratic Discriminant Analysis) yields the best result with an average accuracy of 86.77 %. In **Feature selection by Firefly algorithm**, NLR combined with XGboost achieves the highest accuracy of 88.97 % on average. In **Feature selection by Elephant Herding Optimization (EHO),** DCT combined with SVM (RBF) achieves the highest average accuracy of 94.85 %.

**Table 9:** Classifiers' optimal average performance with and without feature selection for prostate data.

Approaches	Best Methodology		Overall Average accuracy among the classifiers
	Feature Extraction with Classifiers	Accuracy %	
Without Feature Selection	DCT + XGboost	86.03	69.02
Feature selection by Harmonic Search	NLR + QDA	86.77	73.16
Feature selection by Firefly	NLR + XGboost	88.97	75.07
Feature selection by EHO	DCT + SVM(RBF)	94.85	79.19

**Fig. 7:**
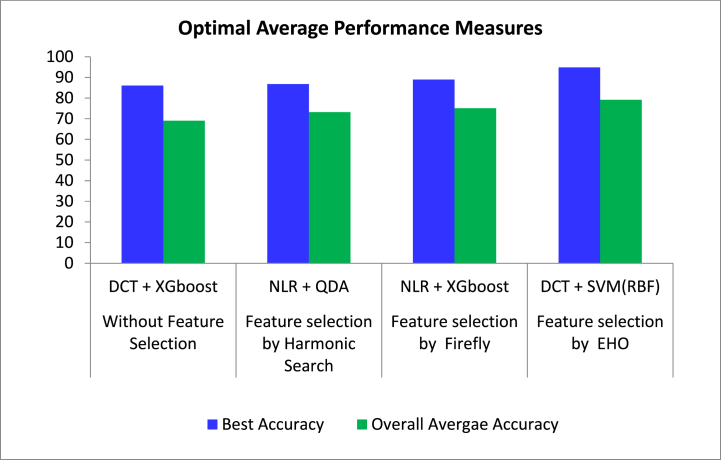
Optimal average performance measures.

DCT with SVM (RBF) outperforms well among the other classifiers. The best accuracy with feature selection by EHO using DCT + SVM (RBF) is 94.85 %, with an overall average accuracy of 79.19 % among the other classifiers. The performance chart is depicted in Fig. 7.

Overall, the EHO-based feature selection approach shows the best performance, with an average accuracy of 79.19 % across all methodologies. This indicates the effectiveness of EHO in selecting relevant features for improving classification accuracy in prostate cancer detection.

**Fig. 8:**
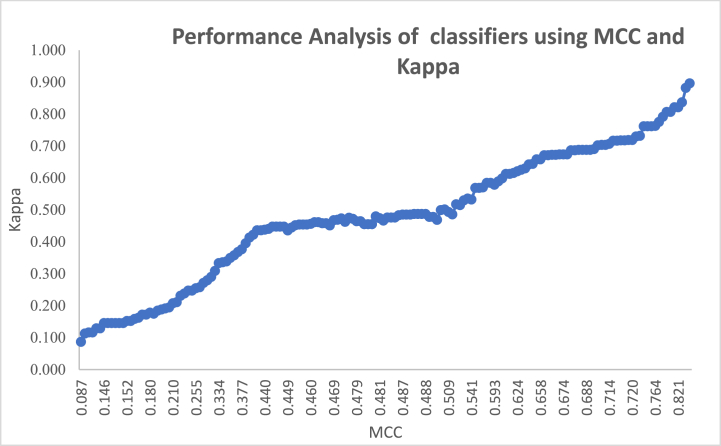
Significance of MCC and Kappa parameters performance of classifiers for different dimensionality reduction techniques.

The two significant parameters that exhibit the hidden performance of the classifiers are MCC and Kappa. Fig. 8 illustrates the significance of the MCC and Kappa parameters' performance of classifiers for five-dimensionality reduction techniques. The figure shows that the MCC and Kappa values closely follow each other in a linear fashion. The average slope for the MCC vs Kappa plot indicates the type of relationship between MCC and Kappa.

It can be observed from Fig. 8 that the MCC values are segmented into three regions. Region 1 ranges from 0 to 0.3, indicating that MCC and Kappa are almost linearly related. In Region 2, MCC values range from 0.31 to 0.5, indicating a deeply varying nonlinear relationship between MCC and Kappa values. In Region 3, MCC values range from 0.6 to 0.7, showing a linear relationship between MCC and Kappa values. Therefore, the significance of classifiers' performance is determined by estimating the average slope from the MCC vs. Kappa values plot. The classifiers with MCC and Kappa values falling in Region 3 are associated with better performance.

**Fig. 9:**
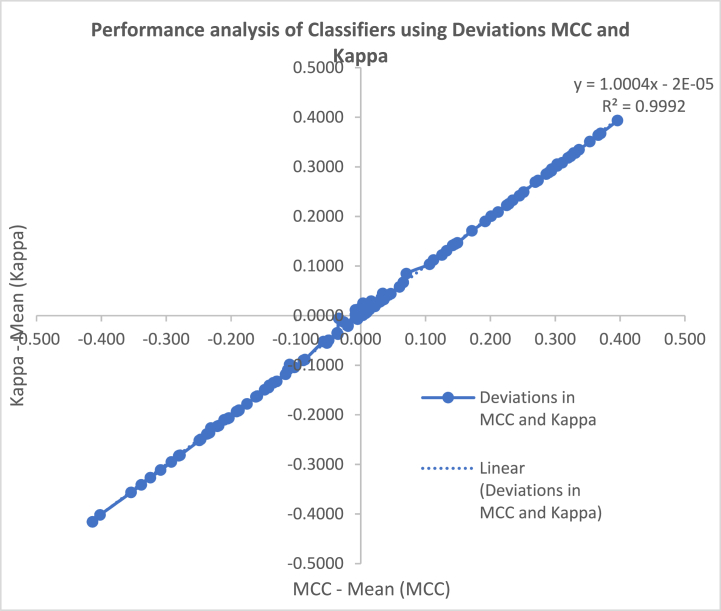
Performance analysis of classifiers using deviations of MCC and Kappa vs Mean (MCC) and Mean (Kappa).

Fig. 9 shows the performance analysis of classifiers using deviations of MCC and Kappa vs. Mean (MCC) and Mean (Kappa) for five dimensionality reduction techniques. Fig. 9 depicts that −0.1 to 0.1 of Kappa values and −0.1 to – 0.1 of MCC values indicate a deeply varying non linear relationship among MCC and Kappa values. Other regions shows a linear relationship among MCC and Kappa values with a slope of 0.999.

Table 10 presents a summary of previous research studies on the detection of Prostate Cancer (PCa) using microarray gene data. The studies were conducted using the TCGA dataset, with some limitations, such as a limited clinical information, a small sample size, and imbalanced data. Different methodologies were used, including correlation, ANOVA, SVM, RF, GA, LASSO, PCA, and SVM, and the performance of each methodology was evaluated in terms of accuracy, AUC, sensitivity, and specificity. The results show that the highest accuracy achieved was 93.6 % using GA and SVM, and the highest AUC achieved was 0.895 using LASSO and SVM. However, some studies had limitations such as small sample size or limited clinical information. The methodology used in this research work gives 94.85 % as high accuracy for the SVM(RBF) classifier with DCT features using the EHO feature selection method.

**Table 10:** Comparison of Previous work related Prostate cancer classification using Microarray gene data.

Author	Year	Dataset	Limitations	Methodology	Result
Saini et al. [41]	2021	GSE6099, GSE10645	Limited number of samples, different platforms	PCA and SVM	Accuracy: 94.16 %
Hassan et al. [42]	2020	GSE40272	Small sample size	GLCM and SVM	Accuracy: 94.44 %
Singh et al. [43]	2018	GSE3325	Small sample size	Feature selection and SVM	Accuracy: 91.7 %
Rahman et al. [44]	2018	GSE21034	Limited number of genes	GA and SVM	Sensitivity: 87 %
Sharma et al. [45]	2021	TCGA	Limited clinical information, imbalanced data	Correlation, ANOVA, SVM	Accuracy: 93.1 %
Piao et al. [46]	2020	TCGA	No validation set, limited clinical information	RF, SVM	Accuracy: 93.5 %
Sun et al. [47]	2020	TCGA, GSE46691	Limited sample size, imbalanced data	GA and SVM	Accuracy: 93.6 %
Hu et al. [48]	2019	TCGA	Limited clinical information	SVM and RF	AUC: 0.89
Chen et al. [49]	2019	TCGA	No validation set	Correlation, PCA, SVM	Accuracy: 91.3 %
Li et al. [50]	2019	TCGA	Limited clinical information	LASSO and SVM	AUC: 0.895
Xu et al. [51]	2019	TCGA	Small sample size	PCA and SVM	Accuracy: 89.4 %
Zhang et al. [52]	2018	TCGA	Limited clinical information, no validation set	LASSO and SVM	Sensitivity: 88.5 %
Khurram et al. [53]	2018	TCGA	Small sample size, limited clinical information	PCA and SVM	Accuracy: 89.09 %
Zhang et al. [54]	2023	GSE6919	Small sample size, lack of validation in external cohorts	Comprehensive genomic profiling	AUC: 0.97
Liu et al. [55]	2024	TCGA-PRAD	Lack of functional validation of identified targets, limited availability of clinical data	Integration of genomic data	Accuracy: 95.4 %
Yang et al. [56]	2023	GSE21034	Imbalanced data	XGBoost	F1-score: 0.92
Chen et al. [57]	2024	ICGC-Prostate	Limited sample size, retrospective nature of the study	Support Vector Machines	Specificity: 93.1 %
Proposed methodology	2023	TCGA	Imbalanced dataset	DCT and SVM(RBF) and EHO	Accuracy: 94.85 %

Using microarray gene data for classifying prostate cancer (PCa) has practical benefits. It can improve early detection by identifying high-risk patients earlier. This approach also enables personalized treatment plans based on the genetic profile of an individual's cancer, leading to more effective treatments with fewer side effects. Additionally, it contributes to cancer research by uncovering new genetic markers and pathways associated with PCa, potentially leading to new therapies and improved prognostic tools. Overall, this approach has practical usefulness in improving PCa diagnosis, treatment, and research.

## 5 Conclusion

The objective of this study is to develop a Prostate Cancer (PCa) classification model using microarray gene data. To achieve this, five feature extraction techniques, including EM, NLR, K means, PCA, and DCT, were employed. Statistical parameters such as mean, skewness, variance, kurtosis, and CCA were calculated from the extracted features and used for analysis. Non-linearity and non-Gaussianity were identified using histograms, normal probability plots, and scatter plots, and appropriate feature selection was employed to address these issues. Three feature selection techniques, namely Harmonic Search (HS), Firefly Algorithm (FA), and Elephant Herding Optimization (EHO) were used to predict the highest classification rate. Eight classifiers, including SVM (Linear), SVM (RBF), SVM (Polynomial), Random Forest, Decision Tree, QDA, Adaboost, and XGBoost were employed, and their performance was evaluated based on various metrics such as accuracy, precision, F1score, MCC, FM, error rate, Jaccard metrics, CSI, Gmean, and Kappa. The DCT method was found to have the best accuracy among the feature extraction methods. DCT with SVM (RBF) was found to have the best accuracy of 94.85 % for the EHO feature selection technique. NLR with XGboost gave an 88.97 % accuracy for Firefly feature selection technique, while NLR with QDA gave an 86.77 % accuracy for the Harmonic Search feature selection technique. The best accuracy was achieved with feature selection by EHO using DCT and SVM (RBF), which gave an accuracy of 94.85 % and an overall average accuracy among the classifiers of 79.19 %.

## 6 Future work

Our study successfully categorized prostate cancer using microarray gene data. However, the generalizability of these findings to other cancers or datasets needs further investigation. While our chosen methods for feature selection and classification worked well, alternative approaches might be even more effective. For future research, we plan to explore deep learning models like Convolutional Neural Networks (CNNs) that could potentially improve accuracy. We also aim to apply our algorithm to various cancers and experiment with more advanced feature selection and classification techniques to enhance its overall effectiveness and applicability. The study suggests that microarray gene data is suitable for deep learning models such as CNN, DNN, VGG-16, and Resnet due to its large size. The proposed algorithm can also be applied to other microarray gene data for cancer prediction, such as cervical and lung cancer, and other engineering problems that involve large datasets.

## Funding

This research was conducted independently without financial support from any funding agencies.

## Data availability statement

The data used in this study, including the counts of healthy samples and malignant prostate cancer samples, as well as the total number of samples, are available upon request from the corresponding author.

## CRediT authorship contribution statement

**Kalaiyarasi Mani:** Writing – original draft, Visualization, Validation, Methodology, Investigation, Data curation, Conceptualization. **Harikumar Rajaguru:** Writing – review & editing, Validation, Supervision, Methodology, Investigation, Conceptualization.

## Declaration of competing interest

The authors declare that they have no known competing financial interests or personal relationships that could have appeared to influence the work reported in this paper.
